# Trait adaptation promotes species coexistence in diverse predator and prey communities

**DOI:** 10.1002/ece3.2172

**Published:** 2016-05-23

**Authors:** Toni Klauschies, David A. Vasseur, Ursula Gaedke

**Affiliations:** ^1^ Department of Ecology and Ecosystem ModelingInstitute for Biochemistry and BiologyUniversity of PotsdamAm Neuen Palais 10D‐14469PotsdamGermany; ^2^ Department of Ecology and Evolutionary BiologyYale UniversityNew Haven, Connecticut06520; ^3^ Berlin‐Brandenburg Institute of Advanced Biodiversity Research (BBIB)D‐14195BerlinGermany

**Keywords:** Coadaptation, equalizing and stabilizing mechanisms, maintenance of functional diversity, niche and fitness differences, supersaturated species coexistence, trait convergence and divergence

## Abstract

Species can adjust their traits in response to selection which may strongly influence species coexistence. Nevertheless, current theory mainly assumes distinct and time‐invariant trait values. We examined the combined effects of the range and the speed of trait adaptation on species coexistence using an innovative multispecies predator–prey model. It allows for temporal trait changes of all predator and prey species and thus simultaneous coadaptation within and among trophic levels. We show that very small or slow trait adaptation did not facilitate coexistence because the stabilizing niche differences were not sufficient to offset the fitness differences. In contrast, sufficiently large and fast trait adaptation jointly promoted stable or neutrally stable species coexistence. Continuous trait adjustments in response to selection enabled a temporally variable convergence and divergence of species traits; that is, species became temporally more similar (neutral theory) or dissimilar (niche theory) depending on the selection pressure, resulting over time in a balance between niche differences stabilizing coexistence and fitness differences promoting competitive exclusion. Furthermore, coadaptation allowed prey and predator species to cluster into different functional groups. This equalized the fitness of similar species while maintaining sufficient niche differences among functionally different species delaying or preventing competitive exclusion. In contrast to previous studies, the emergent feedback between biomass and trait dynamics enabled supersaturated coexistence for a broad range of potential trait adaptation and parameters. We conclude that accounting for trait adaptation may explain stable and supersaturated species coexistence for a broad range of environmental conditions in natural systems when the absence of such adaptive changes would preclude it. Small trait changes, coincident with those that may occur within many natural populations, greatly enlarged the number of coexisting species.

## Introduction

Hutchinson's famous paradox of the plankton questions how a large number of phytoplankton species can coexist in a rather homogeneous environment while all compete for the same few limiting resources (Hutchinson 1961). Since then, many potential mechanisms have been identified to promote species coexistence in time and space including resource partitioning, endogenous consumer–resource cycles, imperfect prey selectivity of predators, and temporal changes in the physical environment (Tilman et al. 1982; Abrams and Holt 2002; Angert et al. 2009; Ryabov et al. 2015).

According to contemporary theory, species coexistence depends on a balance between niche and fitness differences among species (Chesson 2000). Niche differences stabilize coexistence by intensifying intraspecific competition relative to interspecific competition whereas species‐level average fitness differences promote competitive exclusion of less favored species (Tilman 1990; Chesson 2000). Stable coexistence requires that stabilizing niche differences are strong enough to offset the effect of fitness differences (HilleRisLambers et al. 2012). This inequality results in negative frequency‐dependent selection so that each species is released from overall competition when rare (Chesson and Kuang 2008). For example, an inferior and a superior resource competitor (indicating fitness differences) may coexist due to selective predation on the dominant one (imposing niche differences) as this provokes that each species is either limited by resources or predators (Holt 1977; Leibold 1996; Chase et al. 2002). All processes which reduce fitness differences decrease the extent of niche differentiation necessary for coexistence and slow down the rate of competitive exclusion (Hubbell 2005; Holt 2006; Adler et al. 2010).

Current theory of species coexistence mainly assumes species to have distinct and time‐invariant trait values (Tilman et al. 1982; Abrams 1998; Adler et al. 2007), thereby ignoring the potential impact of trait adaptation on coexistence in species rich communities. However, individual species are able to adjust their mean trait values in response to selection on timescales concurrent with changes in their population densities via adaptive evolution or adaptive phenotypic plasticity (Abrams and Matsuda 1997; Hairston et al. 2005; Abrams 2010; Cortez 2011). Such trait adaptation promoted coexistence in consumer–resource models (Abrams 2006a; Lankau and Strauss 2007; Vasseur et al. 2011; Mougi 2013), by enhancing stabilizing niche differences or reducing destabilizing fitness differences among species. For instance, trait adaptation in resource utilization traits reduced the fitness differences between two competitors by allowing for trait convergence (Fox and Vasseur 2008). Furthermore, trait changes within a generalist species stabilized its coexistence with two specialist species in a consumer–resource (Abrams 2006c) and a predator–prey system (Yamamichi et al. 2011) by promoting recurrent cycles in the limiting factors in which either the generalist or the specialists were favored. This strengthened temporal niche differentiation among species. However, these studies make two critically assumptions which are unlikely to be realistic in nature: they restricted trait adaptation to one trophic level and assumed that species could adapt their trait values along the entire trait axis of the community.

First, restricting trait adaptation to one trophic level neglects the potential of prey and predator species to mutually adjust their trait values in response to each other (Kishida et al. 2006; McGhee et al. 2013). For instance, prey species may change their size in response to altered predation pressure to reduce their grazing losses (Kuhlmann and Heckmann 1985; Bergkvist et al. 2012; Gilbert and McPeek 2013). To counteract prey defenses and thus to avoid long periods of food shortage, grazers may also adjust their size or feeding behavior (Kopp and Tollrian 2003; Kishida et al. 2006; Tirok and Gaedke 2007). This may provoke coadaptation in defensive and offensive strategies of prey and predators that may strongly influence the stability and the shape of their dynamics (Abrams 1986; Dercole et al. 2006; Mougi 2012a; Cortez and Weitz 2014). However, its influence on coexistence of predator species and prey species is still unknown.

Second, assuming that species are able to adapt their trait values along the entire trait axis of the community disregards that species generally differ in their functional traits (McGill et al. 2006) and thus their abilities to cope with different environmental conditions including the relative and absolute abundances of other species. In general, interspecific trait variation strongly exceeds intraspecific trait variation (Albert et al. 2010; Auger and Shipley 2013). The latter is constrained by various factors including a lack of genetic variation, developmental constraints, genetic correlations, or costs of plasticity (Smith et al. 1985; Blows and Hoffmann 2005; Kellermann et al. 2009; Murren et al. 2015). Hence, trait changes occurring within ecological time should be restricted to species‐specific limits.

In line with classical niche theory, interspecific trait variation and trade‐offs between ecologically important traits may result in niche differences that stabilize coexistence as different species are favored at different times and locations (Taper and Case 1985; Tilman 2004; Violle and Jiang 2009; Kraft et al. 2015). For instance, energy and resources can be used either to increase reproduction or resistance leading to a trade‐off between strategies maximizing growth and minimizing losses. In this case, coexistence is stabilized by temporal niche differences as the fast‐growing prey is favored at low and the defended prey at high predator densities. In contrast, according to neutral theory (Hubbell 2005; Adler et al. 2007) species coexistence may be promoted by the ecological equivalence of species as less stabilizing mechanisms are needed (Fox and Vasseur 2008). Ecological equivalence likely corresponds to a high trait similarity among species (Vergnon et al. 2009; Violle et al. 2012). Therefore, coadaptation may promote coexistence by allowing species of the same trophic level to be more similar (neutral theory) or dissimilar (niche theory). This convergence (increasing equalizing forces) and divergence (increasing stabilizing forces) of traits may strongly depend on the species' ecological feasible ranges of trait adaptation.

In addition, the impact of trait adaptation on species coexistence may strongly depend on its speed (Abrams 2006b; Mougi 2013). Increasing the speed of trait adaptation may reduce the time‐lag in trait adjustments toward the currently favored trait value which generally promotes species coexistence (Abrams 2006c; Vasseur et al. 2011). However, fast trait changes may also promote biomass oscillations and thus stochastic extinction (Schreiber et al. 2011; Tien and Ellner 2012).

Hence, in this study, we investigate the combined influence of the range and the speed of trait adaptation on species coexistence in a multispecies predator–prey system. In accordance with previous work by Tirok and Gaedke (2010) and Bauer et al. (2014), we assumed the prey species to vary in their intrinsic growth rates and vulnerabilities to predation, while predator species differed in respect to their prey selectivity and ability to graze efficiently on low prey densities. We explicitly consider temporal changes in the trait values of all prey and predator species, thereby allowing for coadaptation between species at the same trophic level and for coadaptation between adjacent trophic levels. We also account for niche differences among species by restricting trait adaptation to species‐specific ecologically feasible ranges.

We show that a sufficiently large and fast potential for trait adaptation as it generally exists in natural communities strongly promoted species coexistence. Coexistence was generally stable when trait adaptation was restricted to a subset of the entire trait space and rather neutrally stable and thus sensitive to stochastic but not to deterministic extinction when all species could attain almost the same trait values.

## Methods

### Description of the multispecies predator–prey model

**Figure 1:**
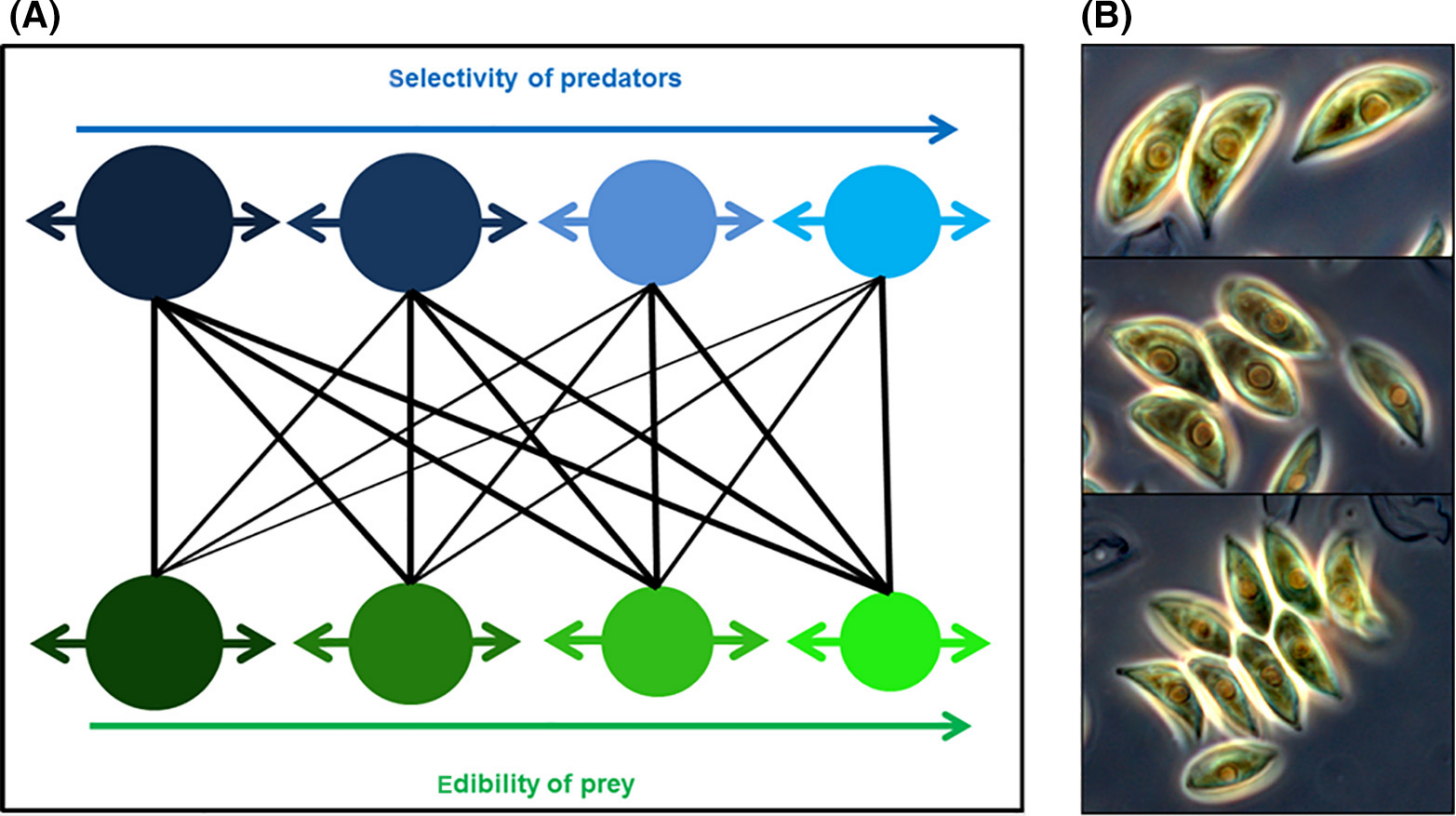
(A) Feeding interactions in the predator–prey system: four prey (bottom; green) and four predator (top; blue) species differ in their edibility (*φ*) and selectivity (*ω*), respectively, increasing from left to right as also indicated by the size of the circle. The thickness of the lines corresponds to the degree of the preference *q*_*i,j*_. The latter depend on both, *φ* and *ω*, and are thus also subject to changes of the mean trait values *φ* and *ω* (indicated by horizontal arrows). (B) Intraspecific size (trait) variation in phytoplankton species because of colony formation. The species shown is *Acutodesmus obliquus* and forms colonies of 2, 4 and 8 cells which may enable an adaptation to altered grazing pressure by changing its effective cell size.

**Figure 2:**
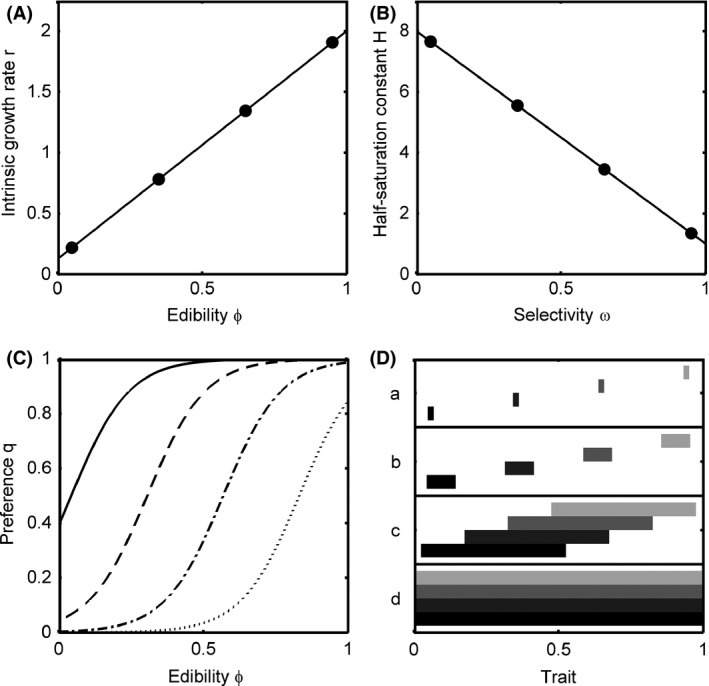
(A) Trade‐off between the intrinsic growth rate *r* and edibility *φ* of the prey species. (B) Trade‐off between the half‐saturation constant *H* and selectivity *ω* of the predator species. The dots mark the center of the four prey and four predator niches along their trait axes. (C) Preference *q* in dependence of *φ* for *ω *= 0.05 (solid), *ω *= 0.35 (dashed), *ω *= 0.65 (dashed‐dotted) and *ω *= 0.95 (dotted). (D) Width of the ranges of trait adaptation of the four species (represented by different shades of gray) that increase from low [(a) 0.02, (b) 0.1] to high [(c) 0.5, (d) 1] values of *w* (cf. methods).

Based on previous studies (Tirok and Gaedke 2010; Tirok et al. 2011; Bauer et al. 2014), we use a modification of the Rosenzweig and Macarthur (1963) model with an extension to multiple prey types (Murdoch 1973). The model contains *S* predator and *S* prey species that differ in their selectivity and edibility, respectively (Fig. 1). To investigate the influence of the range and the speed of trait adaptation on species coexistence, we allow the mean trait values of the individual prey and predator species to change in response to selection. The biomass dynamics of the *i‐*th prey (*P*_*i*_) and the *j*‐th predator (*C*_*j*_) species are described by the following equations:

(1) $$\frac{dP_{i}}{dt}=r_{i}\cdot \left(1-\left(\sum_{z=1}^{S}P_{z}\right)\cdot K^{-1}\right)\cdot P_{i}-\sum_{j=1}^{S}g_{i,j}\cdot C_{j}$$

(2) $$\frac{dC_{j}}{dt}=\left(e\cdot \sum_{i=1}^{S}g_{i,j}-d\right)\cdot C_{j}$$

where *r*_*i*_ is the intrinsic growth rate of the *i*‐th prey species, *K* is the common carrying capacity of the prey community, *e* is the conversion efficiency, and *d* is the per capita death rate of the predators. Foraging on prey *i* by predator *j* is defined by the per capita grazing rate *g*_*i,j*_ for which we assume a type II functional response:

(3) $$g_{i,j}=\frac{g_{\max}\cdot q_{i,j}\cdot P_{i}}{\sum_{z=1}^{S}q_{z,j}\cdot P_{z}+H_{j}}$$

where *g*_max_ is the maximum per capita grazing rate of all predator species and *H*_*j*_ is the half‐saturation constant of the *j*‐th predator species. The interaction between the *i*‐th prey and *j*‐th predator is determined by the preference *q*_*i,j*_ depending on the species‐specific edibility of the prey, *φ*_*i*_, and on the species‐specific selectivity of the predator, *ω*_*j*_, both ranging between 0 and 1 (Figs. 1, 2A and B).

(4) $$q_{i,j}={\left(1+e^{-b\cdot \left(\varphi_{i}-c\cdot \omega_{j}\right)}\right)}^{-1}$$

The preference *q*_*i,j*_ increases with decreasing values of *ω*_*j*_ and increasing values of *φ*_*i*_ (Fig. 2C); that is, nonselective predators (*ω*_*j*_ ≈ 0) have high *q*_*i,j*_ values for all prey species whereas more selective ones (*ω*_*j*_ ≫ 0) have high *q*_*i,j*_ values only for a more restricted prey spectrum (*φ*_*i*_ ≫ 0) which is quantified by *c*. The value of *b* determines the sharpness of the transition of the *q*_*i,j*_ values from nonpreferred to preferred prey species. We set *b *=* *10 which generates a sharp cutoff at the edge of the preferred edibility range in agreement with the “zero‐one rule” established by optimal foraging theory (Krebs 1980).

We assume the intrinsic growth rate of the prey (*r*_*i*_) to trade off linearly with its edibility (*φ*_*i*_) (cf. Leibold 1996; Norberg 2004; Fine et al. 2006) and the half‐saturation constants (*H*_*j*_) of the predators to trade off linearly with their selectivity (*ω*_*j*_) (cf. Tessier et al. 2000; Straub et al. 2011) (Fig. 2A and B):

(5) $$r_{i}=\left(r_{\max}-r_{\min}\right)\cdot \varphi_{i}+r_{\min}$$

(6) $$H_{j}=-\left(H_{\max}-H_{\min}\right)\cdot \omega_{j}+H_{\max}$$

That is, the most edible prey species (*φ*_*i*_ **= 1) has an intrinsic growth rate of *r*_max_ and the least edible prey species (*φ*_*i*_ **= 0) of *r*_min_. Similarly, the maximum (*H*_max_) and minimum (*H*_min_) half‐saturation constants correspond to the food demand of the least (*ω*_*j*_ *= *0) and most (*ω*_*j*_ *= 1*) selective predator species in the model, respectively. Hence, high food selectivity is connected with the ability to maintain positive net‐growth at low prey densities.

The individual prey and predator species are able to change their edibility (*φ*_*i*_) and selectivity (*ω*_*j*_) within species‐specific limits in response to altered environmental conditions to increase their per capita net‐growth rates. These changes were modeled using a general description for selection on a quantitative trait (Lande 1976; Abrams et al. 1993; Abrams 2010):

(7) $$\frac{d\varphi_{i}}{dt}=v\cdot \left(\frac{\partial R_{P_{i}}}{\partial \varphi_{i}}+B\left(\varphi_{i},\Phi_{i}\right)\right)$$

(8) $$\frac{d\omega_{j}}{dt}=v\cdot \left(\frac{\partial R_{C_{j}}}{\partial \omega_{j}}+B\left(\omega_{j},\Omega_{j}\right)\right)$$

where *R*_*Pi*_ **= *(1/P*_*i*_ *)*·*dP*_*i*_ */dt* and *R*_*Cj*_ **= *(1/C*_*j*_ *)*·*dC*_*j*_ */dt* are the per capita net‐growth rates of the *i*‐th prey and *j*‐th predator species. We extended the Geber‐Price method (Hairston et al. 2005) to multispecies communities to show that the parameter *v* scales the speed of trait adaptation relative to the species' biomass dynamics (Appendix S1). Although the approach of quantitative genetics has been used primarily for traits with a genetic basis (Lande 1982), it may also be used to account for changes in the mean trait value via adaptive phenotypic plasticity (Abrams 2010). In this case, *v* may not only depend on the heritable additive genetic variance or mutation rate within a species' population (Lande 1982; Dieckmann and Law 1996), but also on the speed of an individual's plastic response to selection (Abrams and Matsuda 2004; Mougi and Iwasa 2011). Hence, *v* expresses the potential for a response to a selective pressure leading to an adaptive or plastic response. In our model, values of *v *>* *0.25 can only arise in the presence of adaptive phenotypic plasticity as the additive genetic variance cannot exceed this value under our model constraints (0 < *φ *< 1 and 0 < *ω *< 1). For the sake of brevity, we herein refer to *v* as the speed of trait adaptation (cf. Mougi 2012b). The boundary function *B* restricts trait adaptation to the species' ecological feasible range (i.e., its niche) by ensuring that *dφ*_*i*_ /dt and $d\omega_{j}/dt$ strongly increase or decrease when *φ*_*i*_ and *ω*_*j*_ approach their lower (*φ*_*i,*min_ = (1−*w*)*·Φ*_*i*_) or upper (*φ*_*i,*max_ = (1−*w*)*·Φ*_*i*_+*w*) limits, respectively (cf. Abrams and Matsuda 2004; Abrams 2010):

(9) $$B\left(\varphi_{i},\Phi_{i}\right)=-\tan\left(\frac{\pi }{2}\cdot {\left(\frac{2}{w}\cdot \left(\varphi_{i}-\left(\Phi_{i}-w\cdot \left(\Phi_{i}-0.5\right)\right)\right)\right)}^{\left(2\cdot s+1\right)}\right)$$

(10) $$B\left(\omega_{j},\Omega_{j}\right)=-\tan\left(\frac{\pi }{2}\cdot {\left(\frac{2}{w}\cdot \left(\omega_{j}-\left(\Omega_{j}-w\cdot \left(\Omega_{j}-0.5\right)\right)\right)\right)}^{\left(2\cdot s+1\right)}\right)$$

The parameters *Φ*_*i*_ and *Ω*_*j*_ determine the locations of the prey and predator niches along their trait axes and thus refer to general niche differences among species (Fig. 2). The width of the species' niches and thus their accessible ranges of trait adaptation are determined by the parameter *w* (Fig. 2D). For *w *=* *0, species are not able to change their trait values in response to selection, whereas for *w *=* *1, all species share the same range of trait adaptation. The parameter *s* determines the steepness of *B* at the edges of the species' trait range. A more detailed discussion of equations (7) and (8) is given in Appendix S2 and in Abrams (2010).

### Numerical simulations

We conducted numerical simulations of our model for different values of *w* and *v* in which we allowed (extinction study) or prevented species extinction (invasion study). The first approach enables the investigation of the extent and stability of species coexistence by recording the final richness, that is, the number of species surviving until the end of the simulation, the presence of long‐term trends in the species biomass dynamics, and their sensitivity to environmental noise. Coexistence is expected to be stable if the biomass dynamics exhibit no long‐term trends and rather low sensitivities to environmental noise. The second approach reveals the stabilizing and equalizing mechanisms crucial for species coexistence.

### Extinction study

We simulated a full‐factorial combination of 31 values of the speed *v* ([10^−3^,10^−2.9^,…,10^0^]) and 21 values of the range *w* ([10^−2^,10^−1.9^,…,10^0^]) of trait adaptation for a system with initially four prey and four predator species (cf. Fig. 1A). We assumed a regular spacing of the values of *Φ*_*i*_ and *Ω*_*j*_ along the respective trait axes with *Φ*_*1*_ and *Ω*_*1*_ equal to 0.05 and *Φ*_*4*_ and *Ω*_*4*_ equal to 0.95 representing a high niche differentiation among species in the absence of trait adaptation. Initial trait values *φ*_*i*_ and *ω*_*j*_ were set equal to the species‐specific constants *Φ*_*i*_ and *Ω*_*j*_. To generalize the results, we also simulated systems with 16 prey and 16 predator species for *w *=* *0.2 and for three different values of *v* (10^−1.5^, 10^−1^, 10^−0.5^).

We parameterized our model in accordance with previous studies (Table 1; Tirok and Gaedke 2010; Tirok et al. 2011; Bauer et al. 2014) for planktonic systems consisting of phytoplankton and their ciliate predators (Hansen et al. 1997; Tirok and Gaedke 2007). We kept the initial total biomass of prey and predators constant at *K*/2 and *K*/6, respectively, but varied the initial distributions of species' biomasses in five ways: even across species, decreasing and increasing linearly along the trait axes, and negative and positive parabolic distributions. The resulting 25 different initial conditions (five for the prey, five for the predator) allowed us to capture potential variation in the final species composition. Each simulation lasted for 10^5^ time units. We assumed species as extinct and set their biomasses to zero if their biomasses dropped below 10^−9^ of the carrying capacity *K*.

**Table 1:** State variables and parameters used in the model following Tirok and Gaedke (2010). The parameters are inspired by considering the biomasses in units of carbon in the upper most 20 m of the water column of Lake Constance corresponding approximately to the euphotic zone and the epilimnion (Tirok and Gaedke 2007). Hence, the units g/m^2^ of biomasses refer to the biomass in the water column of the upper meters (m·g/m^3^ = g/m^2^)

	Description	Unit	Value
Variables			
Biomasses			
P	Prey biomasses	g C/m^2^	–
C	Predator biomasses	g C/m^2^	–
Traits			
*φ*	Mean edibility of prey	–	–
*ω*	Mean selectivity of predators	–	–
Parameters			
Biomasses			
*K*	Common carrying capacity	g C/m^2^	10
*H*_max_	Maximum half‐saturation constant	g C/m^2^	8
*H*_min_	Minimum half‐saturation constant	g C/m^2^	1
*et*	Extinction threshold = minimum biomass	g C/m^2^	10^−8^
Rates			
*d*	Death rate of predators	day^−1^	0.15
*g*_max_	Maximum grazing rate of predators	day^−1^	2
*r*_max_	Maximum growth rate of prey	day^−1^	2
*r*_min_	Minimum growth rate of prey	day^−1^	0.25
Traits			
*w*	Potential range of trait adaptation	–	0.01–1
*v*	Speed of trait adaptation	–	0.001–1
Scaling			
*e*	Conversion efficiency of predators	–	0.3
*s*	Steepness of the boundary function	–	10
*c*	Scaling of the preference function	–	7/8
*b*	Steepness of the preference function	–	10

To distinguish between stable coexistence and prolonged co‐occurrence, we evaluated the presence of long‐term trends in the species biomass dynamics which indicate prolonged transients and ongoing competitive exclusion (Chesson 2000). We estimated the long‐term trends for systems showing at least some biomass variation in time (CV* *> 10^−3^) by calculating the Pearson's correlation between log_10_ biomass and time, using the last 10^4^ time steps. We evaluated the significance of the correlation coefficients by comparing their values against a null distribution of 100 correlation coefficients that were obtained from randomized time series of biomasses (*P *<* *0.05).

To further distinguish between stable and neutrally stable coexistence, we tested for the sensitivity of the species biomass dynamics to environmental noise by continuing the simulations for six parameter combinations of *w* ([0.1, 1]) and *v* ([10^−1.5^,10^−1^,10^−0.5^]) with and without noise. In both cases, we started the simulations with the final state of the previous model runs. In the second case, we additionally added multiplicative white noise to the biomass dynamics to mimic environmental stochasticity (cf. eqns. 1 and 2 and Braumann 2008):

(11) $$\frac{dP_{i}}{dt}=r_{i}\cdot \left[1-\left(\sum_{z=1}^{S}P_{z}\right)\cdot K^{-1}\right]\cdot P_{i}-\sum_{j=1}^{S}g_{i,j}\cdot C_{j}+n\left(0,\varepsilon \right)\cdot P_{i}$$

(12) $$\frac{dC_{j}}{dt}=\left(e\cdot \sum_{i=1}^{S}g_{i,j}-d\right)\cdot C_{j}+n\left(0,\varepsilon \right)\cdot C_{j}$$

The random numbers were drawn independently from a normal distribution with mean and standard deviation equal to 0 and ԑ for each time step and differential equation prior to the numerical integration. We run 25 replicates of stochastic simulations for 10^5^ time steps using two different values of ԑ (0.05 and 0.005) and compared their average final richness to the final richness obtained without disturbance. Environmental stochasticity is expected to promote stochastic extinctions in case of neutrally stable coexistence but not in case of stable coexistence.

Finally, we evaluated a potential clustering of species in the trait space by discretizing the trait axis into functional groups, each of which had a width equal to 0.01.

### Invasion study

To reveal the causes underlying the pattern in final richness, we assessed the effects of *w* and *v* on stabilizing and equalizing mechanisms acting within the prey community. First, we estimated time‐averaged niche differences (ND) and fitness differences (FD) among prey species by employing two indices given in Carroll et al. (2011):

(13a) $$\text{ND}=1-\prod_{k=1}^{S}\sqrt[{S}]{s_{k}}$$

(13b) $$\text{FD}=\exp\left[{\left(\frac{1}{S}\sum_{k=1}^{S}{\left(\ln s_{k}\right)}^{2}-{\left(\frac{1}{S}\sum_{k=1}^{S}\ln s_{k}\right)}^{2}\right)}^{\frac{1}{2}}\right]-1$$

with

(13c) $$s_{k}:=\frac{I_{k,a}-I_{k,p}}{I_{k,a}}$$

The index *s*_*k*_ describes the standardized difference between the invasion growth rates (*I*) of the *k*‐*th* prey species in the absence (*I*_*k,a*_) and presence (*I*_*k,p*_) of its resident community. This index thus represents the species' sensitivity to interspecific competition such as direct resource or predator‐mediated, apparent competition (Carroll et al. 2011). An invader with substantial niche differences to the resident community experiences weak negative effects from interspecific competition, keeping *I*_*p*_ close to *I*_*a*_ and *s*_*k*_ small. In contrast, small niche differences yield strong competition among species which reduces *I*_*p*_ and increases *s*_*k*_. Large fitness differences (FD) imply that the effect of interspecific competition on the fitness of a focal species varies greatly among species whereas small fitness differences (FD) arise when all species experience a similar intensity of interspecific competition. In contrast to Carroll et al. (2011), we subtract one from the main expression in equation ((13b)) so that FD is zero when all species have equal fitness.

We calculated *I*_*a*_ for the prey analytically using their intrinsic growth rates in monoculture at their maximum edibility. To determine *I*_*p,*_ we set the biomass of the invading prey species to zero which prevents actual growth in its biomass but we still allowed its trait value to change in response to selection. To prevent the exclusion of the resident species, we added a small immigration rate (*I *= 10^−8^) to equations (1) and (2) describing the prey and predator biomasses dynamics. We conducted these simulations by following a bifurcation approach which allows us to stick to a certain attractor of the species composition. Hence, we initially ran the model for 10^5^ time steps with low values of *w* (0.01) and *v* (10^−1.5^) and then used the final values to initialize runs at slightly higher parameter values, iterating this process across a range of *w* ([10^−2^, 10^−1.97^,…, 10^0^]). We estimated *I*_*p*_ from the last 10^4^ time steps.

This approach is based on the invasibility criterion, stating that prey species stably coexist if all are able to increase from low densities in the presence of their competitors, that is, if intraspecific competition is larger than interspecific competition at low density (Chesson 2000). Furthermore, when applied to nonequilibrium dynamics, the invasibility criterion relies on temporal averaging and therefore ignores niche differences that may arise only during critical temporary periods. Hence, we also assessed temporal niche differences among prey species based on pairwise temporal correlations between their mean trait values using the last 10^4^ time steps of simulations where all species were kept in the system by adding a small immigration rate to equations (1) and (2). Positive correlations between trait dynamics indicate that species respond very similar to environmental changes whereas negative correlations indicate differences in response.

Simulations and analyses were performed in MATLAB, version 7.13, using solver ode23 for ODEs (The MathWorks Inc., Natick, MA, 2011). We increased the precision of the solver by reducing the absolute and relative tolerance to 10^−10^ and 10^−8^ and the maximum step size to 0.001.

## Results

**Figure 3:**
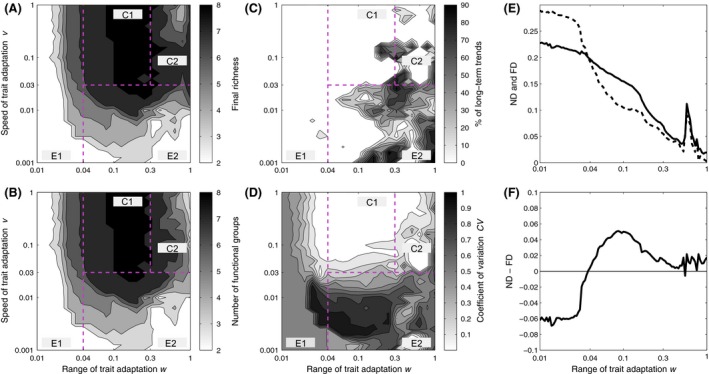
(A) Summed final prey and predator species richness, (B) summed number of prey and predator functional groups, (C) percentage of remaining prey and predator species exhibiting long‐term trends in their biomass dynamics and (D) the mean coefficient of variation of prey and predator population dynamics during the last 10,000 time steps averaged over all initial conditions in dependence of the range (*w*) and speed (*v*) of trait adaptation for initially four prey and four predator species. Four regions exhibiting similar patterns are marked: two regions of extinction of numerous or all species (E1, E2) and two regions of species coexistence (C1, C2). (E) Estimated time‐averages of niche (ND *,* solid) and fitness differences (FD *,* dashed) among the four prey species and (F) estimated net‐stabilizing effects based on their difference for *v *=* *0.03 in dependence of the range (*w*) of trait adaptation.

The range (*w*) and speed (*v*) of trait adaptation strongly influenced species coexistence. For brevity, we block the presentation of the results into four regions of the parameter space that exhibit common patterns and discuss their dynamics and underlying mechanisms. In general, small (*w < 0.04*) and slow (*v *<* *0.03) trait adaptation did not promote species coexistence (Fig. 3A, regions E1, E2). A simultaneous increase in *w* and *v* resulted in considerable adjustments of the species' mean trait values that enabled both stable (Fig. 3A, region C1) and rather neutrally stable coexistence (partly very slow exclusion; Fig. 3A, region C2). These general findings are independent of the exact parameter values chosen (see Appendix S3). As final prey and predator richness were highly correlated (*R* ^2^ = 0.89), we jointly consider them.

### Small trait adaptation did not promote species coexistence – Region E1

**Figure 4:**
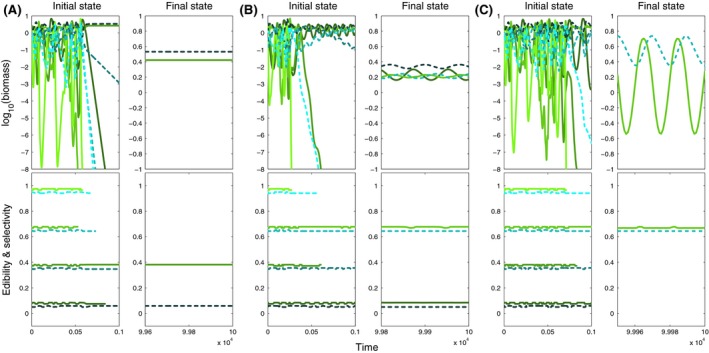
Biomass (top) and mean trait dynamics (bottom) for small ranges (*w *=* *0.01, cf. Fig. 3A, region E1) and (A) low (*v *= 10^−2.5^), (B) moderate (*v *= 10^−1.5^) and (C) high (*v *= 10^−0.5^) speed of trait adaptation, showing the initial (left panels) and final (right panels) state for four prey (colored green) and four predator species (colored blue). Initial edibility and selectivity increase from dark to light shades of colors (cf. Fig. 1). Note the different scales for the two time periods because of differences in the amplitudes and periods of the oscillations.

Small ranges of trait adaptation (*w *<* *0.04) prevented distinct changes in the trait values over time whereas the biomasses showed large amplitude oscillations at the beginning of the simulation leading to rapid exclusion of numerous species and low final richness, irrespective of *v* (Figs. 3A, 4). After 10^5^ time units, typically one predator–prey pair survived showing either oscillatory or stable dynamics depending on the remaining trait values (Figs. 3D, 4). The initial prey and predator species exhibited considerable trait and niche differences as indicated by high values of the related index, ND, where each species occupied its own niche with little overlap to others, stabilizing coexistence (Fig. 3E). However, the species also exhibited large fitness differences as indicated by high values of the index, FD, promoting competitive exclusion (Fig. 3E). According to the difference between ND and FD, the niche differences among species did not stabilize coexistence sufficiently to compensate for their fitness differences giving rise to species extinction (Fig. 3F).

### Slow trait adaptation did not promote species coexistence – Region E2

**Figure 5:**
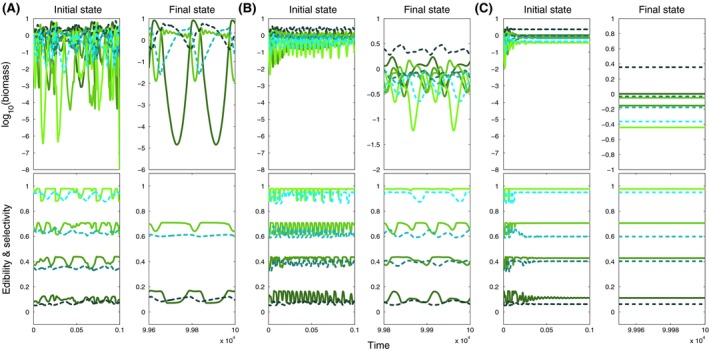
Biomass and mean trait dynamics as in Figure 4 but for moderately large ranges (*w *=* *0.1, cf. Fig. 3A, regions E2 and C1) and (A) low (*v *= 10^−2.5^), (B) moderate (*v *= 10^−1.5^) and (C) high (*v *= 10^−0.8^) speed of trait adaptation.

**Figure 6:**
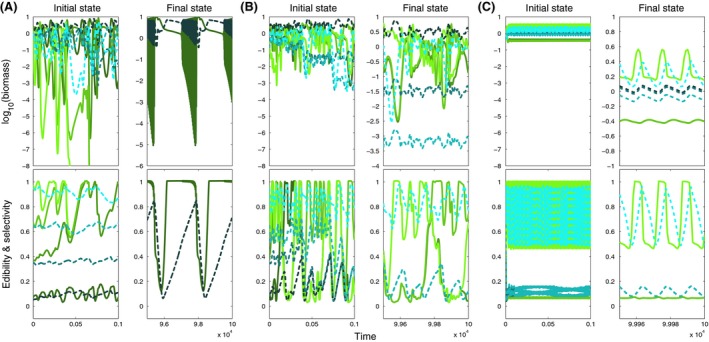
Biomass and mean trait dynamics as in Figure 4 but for large ranges (*w *=* *1, cf. Fig. 3A, regions E2 and C2) and (A) low (*v *= 10^−2.5^), (B) moderate (*v *= 10^−1.5^) and (C) high (*v *= 10^−0.5^) speed of trait adaptation.

Low speed of trait adaptation (*v *<* *0.03) resulted in a temporal mismatch between ecological and evolutionary processes preventing contemporary trait adjustments in response to selection, irrespective of *w*. As in region E1, this led to rapid exclusion of numerous species (Fig. 3A) despite substantial trait changes over time (Fig. 5A). However, 25% of the simulations exhibited supersaturated coexistence, that is, the number of species in one trophic level exceeded the number of species in the other trophic level. In most cases, two predators grazed on a single prey species (cf. Appendix S5). Systems finally comprising only one prey, and one predator species usually showed high frequency biomass oscillations that were superimposed upon low‐frequency trait oscillations (Fig. 6A). This led to periodic regime shifts in the biomass dynamics in which a predominance of fast oscillations alternated with slow ones. The temporally high‐amplitude oscillations within the fast component of the biomass dynamics corresponded to the prolonged occurrence of highly edible prey and highly selective predators with very high intrinsic growth and grazing rates (Fig. 6A). The period of the low‐frequency part in the biomass oscillations strongly exceeded the sampling period and thus promoted the detection of long‐term trends that do not indicate prolonged transients of competitive exclusion in these systems (cf. Figs. 3C, 6A). For larger ranges of trait adaptation (*w > 0.04*), a transition from species poor (Fig. 3A, region E2) to species rich (Fig. 3A, regions C1 and C2) systems occurred at about *v *≈* *0.03, often characterized by irregular dynamics and long‐term trends in species biomasses (Fig. 3C).

### Constrained but fast trait adaptation promoted stable species coexistence – Region C1

Moderate ranges (0.04 < *w *<* *0.3) and a sufficiently high speed (*v *≥* *0.03) of trait adaptation allowed species to make considerable and fast trait adjustments within their distinct ranges (Fig. 2D) delaying or preventing species exclusion (Fig. 5). Higher values of *v* accelerated the changes in traits relative to the changes in biomasses. This promoted stable coexistence of functionally different species as indicated by the rarity of long‐term trends in their biomass dynamics (Fig. 3C, region C1). Depending on *w* and *v,* we found that the biomass and trait dynamics were either at equilibrium or oscillatory (Fig. 3D). At equilibrium, the species composition reached a final state after unique shifts in their trait values whereas in case of oscillations species showed ongoing trait adjustments (Fig. 5B and C).

The time‐averaged niche differences, ND, and fitness differences, FD, among prey species both declined with *w* but the decrease in FD was more pronounced than that of ND. This gives rise to a net increase in stabilizing mechanisms as indicated by FD‐ND (Fig. 3E and F). In addition, for pronounced and regular oscillations, the trait values of the most edible prey cycled out of phase with that of the least edible prey, implying that their trait values became temporally more similar or dissimilar (*correlation coefficient ρ* ≈ −0.75; Fig. 5B). This gave rise to a temporal interplay between niche and fitness differences among prey species that stabilized their coexistence. Coadaptation was essential for coexistence as it ensured that fitness differences did not exceed the effect of niche differences (Fig. 3E). Preventing coadaptation among prey or predator species by assigning constant trait values to some or all of them or assuming only nonadaptive random trait changes strongly reduced the final richness (for details see Appendix S4).

Stable coexistence is further indicated by the low sensitivity of the species biomass dynamics to environmental stochasticity as neither low [final richness = 8 ± 0 (*v *= 10^−1.5^); 8 ± 0 (*v *= 10^−1^); 8 ± 0 (*v *= 10^−0.5^)] nor high [final richness = 7.84 ± 0.55 (*v *= 10^−1.5^); 8 ± 0 (*v *= 10^−1^); 8 ± 0 (*v *= 10^−0.5^)] levels of noise did substantially reduce the final richness below a value of 8, that is, below the final richness obtained in the absence of noise.

**Figure 7:**
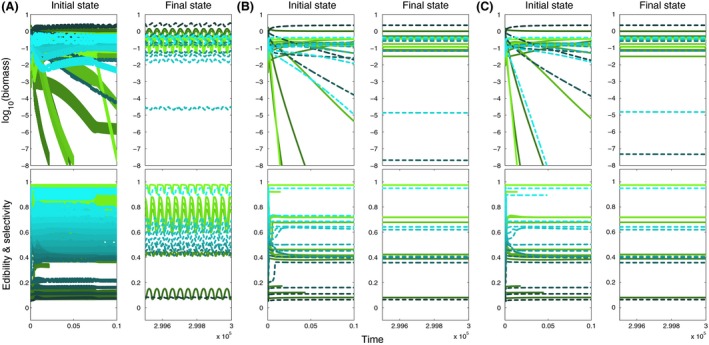
Biomass and mean trait dynamics as in Figure 5 but for initially 16 prey (colored green) and 16 predator species (colored blue) and for different high values of the speed of trait adaptation, (A) *v* = 10^−1.5^, (B) 10^−1^, (C) 10^−0.5^ showing the first 10^4^ (left panels) and last 500 time steps (right panels).

**Figure 8:**
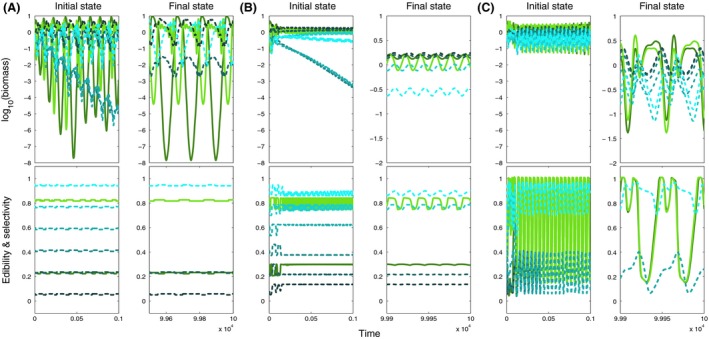
Biomass (top) and mean trait dynamics (bottom) for moderate speed (*v *= 10^−1^) and (A) small (*w *=* *0.01), (B) intermediate (*w *=* *0.1) and (C) large (*w *=* *1) ranges of trait adaptation, showing the initial (left panels) and final (right panels) state for initially two prey (colored green) and six predator species (colored blue). Initial edibility and selectivity increase from dark to light shades of colors (cf. Fig. 1). In contrast to the results shown in Appendix S5, the prey species exhibited intermediate rather than extreme initial trait values. In (A) one of the less selective predators has a negative long‐term trend in its biomass dynamics suggesting prolonged competitive exclusion.

When the trait ranges, that is, niches, of neighboring species overlapped, that is, *w > Φ*_i_‐*Φ*_i−1_ for prey and *w > *Ω_j_‐Ω_j−1_ for predators, species were able to cluster into functional groups with very similar trait values and highly synchronized biomass and trait dynamics. In systems comprising initially 16 prey and 16 predator species, prey and predators formed functional groups that persisted throughout time (Fig. 7). This resulted in high final richness of six prey and 10 predators (*v *= 10^−1.5^), 12 prey and 10 predators (*v *= 10^−1^) and 12 prey and 10 predators (*v *= 10^−0.5^) after 3·10^5^ time steps. Interestingly, the four main functional groups formed within the prey and predator communities, respectively, exhibited very similar trait values to the ones shown for systems with initially four prey and four predator species. This suggests that our model system only allows stable coexistence of up to four prey and four predator species which is supported by the long‐term trends present in the biomass dynamics of the remaining species. However, the very long persistence of many other species indicates strong equalizing mechanisms within functional groups. Indeed, the high trait similarity reduced the fitness differences of species within functional groups while maintaining trait and thus niche differences among species of different functional groups. The unequal number of final prey and predator species in the simulations with initially 16 prey and 16 predator species reveals that trait adaptation may enable supersaturated coexistence. Indeed, trait adaptation strongly promoted stable supersaturated coexistence within the prey or predator community in systems with an unequal initial number of prey and predator species (*S*_prey_ = 6 and *S*_predators_ = 2 or *S*_prey_ = 2 and *S*_predators_ = 6; for details see Appendix S5). The extent of supersaturated coexistence depended on the species' initial trait values. For example, trait adaptation allowed stable coexistence of four predator species on two prey species for *w *=* *0.1 when the prey had initially intermediate (Fig. 8B) rather than extreme trait values (cf. Appendix S5). However, we never observed stable supersaturated coexistence in the absence of trait adaptation irrespective of the initial trait values.

### Large and fast trait adaptation promoted unstable coexistence – Region C2

Similar to region C1, the biomass and trait dynamics were either at equilibrium or oscillatory for higher values of *w* (*>0.3*) and *v* (*>0.03*) depending on their exact values (Fig. 3D). However, in contrast to region C1, species often showed rather complex, irregular and high‐amplitude biomass dynamics (Fig. 6B and C) which frequently exhibited long‐term trends suggesting prolonged transition periods of competitive exclusion (Fig. 3C). This result is supported by very small values of ND and FD and lower final richness (Fig. 3A and E). Hence, the species became functionally redundant sharing almost the same trait space and thus fitness landscape, that is, the per capita net‐growth rate as a function of the trait value, so that the species were able to replace each other (Fig. 2D). The system exhibited rather neutrally stable coexistence which is confirmed by the relatively high sensitivity of the biomass dynamics to environmental stochasticity. Independently of *v*, high levels of environmental noise substantially reduced the final richness below a value of 8 which was the final richness obtained without noise [final richness = 5.88 ± 0.73 (*v *= 10^−1.5^); 4.52 ± 0.96 (*v *= 10^−1^); 5.40 ± 1.22 (*v *= 10^−0.5^)].

**Figure 9:**
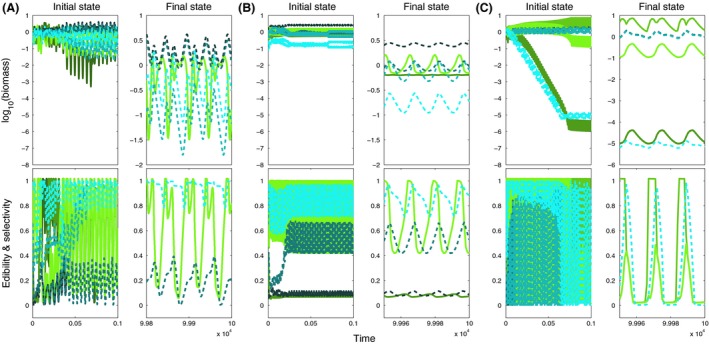
Biomass and mean trait dynamics as in Figure 6 but for different high values of the speed of trait adaptation, (A) *v *= 10^−1^, (B), *v *= 10^−0.7^, (C) *v *=* *1.

For very large ranges of trait adaptation (*w *≈* *1), the four prey and four predator species clustered into functional groups reducing fitness differences among species of the same functional group (compare region C2 of Fig. 3A and B). Such a formation of functional groups was temporally variable for moderate values of *v* (Fig. 6B) and usually persisted throughout time for higher values of *v* (Figs. 6C, 9). The remaining prey species formed 1 or 2 functional groups, whereas the predators split into up to 3 functional groups. If only a single functional group remained, the temporal changes in its mean trait value covered the whole trait space (Fig. 9A and C). In contrast, the individual traits of 2 or 3 remaining prey or predator functional groups partitioned the trait space almost equally (Figs. 6C, 9B). The formation of functional groups in our model occurred consistently and independently of the initial trait values of the species (results not shown).

The clustering of species into different functional groups suggests the maintenance of stabilizing niche differences within the prey and predator communities. This is supported by the fact that low levels of environmental stochasticity were usually not sufficient to reduce the final richness during the simulation period [final richness = 7.72 ± 0.46 (*v *= 10^−1.5^); 8 ± 0 (*v *= 10^−1^); 8 ± 0 (*v *= 10^−0.5^)] below a value of 8. However, in contrast to region C1, coexistence depended much more on the recurrent trait oscillations within a functional group as indicated by the unequal number of prey and predator functional groups giving rise to supersaturated coexistence based on temporal niche differences (Figs. 6, 9). Indeed, 33% of the simulations exhibited supersaturated coexistence, typically with four prey species grazed upon by three predators (cf. Appendix S5). Furthermore, trait adaptation enabled a clustering of species on the trait axes that allowed for supersaturated coexistence of up to six prey and two predator species or two prey and six predator species when running simulations with an unequal initial number of prey and predator species (*S*_prey_ = 6 and *S*_predators_ = 2 or *S*_prey_ = 2 and *S*_predators_ = 6; Fig. 8C; Appendix S5). The increase in final richness demanded higher values of *v* for higher values of *w (w *>* *0.3) than for intermediate values of *w* (0.04 ≤ *w *≤* *0.3) (cf. Fig. 3A) where several prey and predator species frequently survived already with *v *>* *0.003 (Fig. 5A).

## Discussion

Species are able to adjust their traits in response to selection and recent studies showed that such frequently neglected trait adaptation may strongly stabilize population dynamics and species coexistence (Abrams 2000, 2006c; Vasseur et al. 2011; Mougi 2013). However, these studies restricted trait adaptation to one trophic level and allowed species to change their trait values over the entire trait space of the community. The first assumption neglects the potential of prey and predator species to mutually adjust their defensive and offensive strategies to each other (Lankau 2011). The second assumption disregards that species generally differ in their functional traits and thus abilities to cope with different environmental conditions, likely giving rise to stabilizing niche differences (Taper and Case 1992; Tilman 2004). Hence, we used an innovative model approach that allows for simultaneous coadaptation within and among trophic levels to investigate the influence of the range (*w*) and the speed (*v*) of trait adaptation on coexistence in multispecies predator and prey communities.

In general, our results show that narrow and slow trait adaptation led to low final richness whereas sufficiently large and fast trait adaptation yielded higher final richness. Species coexistence was stable when trait adaptation was restricted to species‐specific limits maintaining trait and thus niche differences among species. Species coexistence was rather neutrally stable when all species could attain almost the same trait values preventing strong niche differentiation. We thus demonstrate that coadaptation among prey and predators can lead to recurrent changes in defense and offense traits that provide novel stabilizing and equalizing effects which is in line with theoretical considerations of Lankau (2011) and will be discussed below. We describe our results in terms of species coexistence, but they hold equally well for the coexistence of clones in asexually reproducing populations and thus the maintenance of their genetic diversity.

### Small and slow trait adaptation did not enable species coexistence – Regions E1 and E2

When the range of trait adaptation was strongly constrained (*w *<* *0.04), species exhibited considerable niche differences that promoted coexistence mostly by selective predation on fast‐growing prey and resource partitioning among predators. However, high time‐averaged niche differences implied also high time‐averaged fitness differences. Thus, in the absence of trait adaptation the niche differences were not sufficient to compensate for the large fitness differences yielding fast exclusion of most species. Finally, usually one prey and one predator species survived showing either oscillatory or stable dynamics depending on the remaining prey and predator traits. Cyclic predator–prey dynamics require a sufficiently strong nonlinearity in the predator's functional response so that it reaches half of its maximum at prey densities well below the prey's carrying capacity, *K* (Abrams 2006c). This was given in our model for highly selective predators (half‐saturation constant *H *≈* *1) but not for nonselective predators (*H *≈* *8, *K *=* *10). Our results confirm that trait adjustments which are slow compared to the ecological dynamics are insufficient to promote the maintenance of species‐rich communities (Vasseur et al. 2011; Mougi 2013).

### Constrained and fast trait adaptation promoted stable species coexistence – Region C1

Sufficiently large (0.04 < *w < 0.3*) and fast (*v *≥* *0.03) trait adaptation allowed considerable trait adjustments in response to selection resulting in a strong dampening of the biomass oscillations and stable coexistence of functionally different prey and predators. The prevailing characteristics of the prey community selected for predator traits more suitable for exploiting the dominant prey. The subsequently enhanced grazing pressure on the dominant prey was accompanied by a release in the grazing pressure on rare prey promoting their recovery. In addition, coadaptation among prey species stabilized their coexistence further by allowing the well edible prey to defend themselves against predation and the less edible prey to increase their competitive abilities, both of which increases the fitness of the different prey species at low densities (cf. Appendix S4). These trait and biomass changes in the prey community, in turn, improve the food supply for the rare predators. Hence, coadaptation in defense and offense traits may stabilize coexistence by reducing the strength of pairwise trophic interactions at low densities (cf. Kokkoris et al. 2002; Imura et al. 2003; Bolnick et al. 2011) and enhancing it at high densities. This gives rise to negative frequency dependence that prevents overexploitation of the prey and long periods of starvation of the predators.

Beyond stabilizing coexistence via (temporal) niche differentiation, trait adaptation also equalized species performances in our model by allowing a reorganization of pairwise trophic interactions that alter their strength. For example, highly selective predators were able to broaden their prey spectrum whereas prey species enhancing their growth rate became accessible to more predators. Both promoted an increased connectivity between the two trophic levels that reduced fitness differences. Our result is in line with findings from food web models where the presence of adaptive foragers strongly promoted species persistence when the overall connectivity was sufficiently high (Kondoh 2003; Uchida et al. 2007; Heckmann et al. 2012).

The stabilizing and equalizing effects of trait adaptation in our model are in line with previous model results (Tirok and Gaedke 2010; Bauer et al. 2014) where additional stabilizing and equalizing mechanisms were a priori introduced to ensure coexistence of multiple prey and predator species. Their functional response was similar to a type III functional response, and all predators were able to consume a certain amount of less edible prey buffering more selective predators from extinction when prey composition shifted toward less edible species. As a result, our species biomass dynamics for higher values of *v* and *w* look very similar to those of Tirok and Gaedke (2010) and Bauer et al. (2014). However, in contrast to a functional type III response where the negative frequency dependence arises instantaneously at low densities, the reduction in grazing pressure on rare prey and the enhancement of grazing of rare predators in our model occur with time‐lags that are inversely proportional to *v*. This is in line with previous studies where higher speed of trait adaptation was needed to promote coexistence in consumer–resource models (Abrams 2006c; Vasseur et al. 2011; Mougi 2013).

Our results also show that coadaptation among prey and predator species promoted coexistence by allowing species to cluster into functional groups influencing both stabilizing and equalizing mechanisms at the same time. Coexistence was stabilized by reducing fitness differences among species of the same functional group while maintaining niche differences among species of different functional groups. This result is in line with recent discussions that both niche differences and neutrality jointly act to maintain species rich communities (Bonsall et al. 2004; Vergnon et al. 2009). Hence, two contrasting windows of opportunity exist for species to coexist: being functionally sufficiently different or being sufficiently similar (Scheffer and van Nes 2006). In the first case, weak equalizing mechanisms are compensated for by strong stabilizing mechanisms whereas in the second case strong equalizing mechanisms promote coexistence in the absence of strong stabilizing mechanisms. In line with Scheffer and van Nes (2006), we show that trait adaptation may enable a self‐organization of species' traits on the trait axes that promote coexistence via the formation of functional groups, that is, the generation of stable clusters of similar species on the trait axes. However, in contrast to Scheffer and van Nes (2006), in our model species clusters arose on an ecological timescale enabling species coexistence even without additional stabilizing mechanisms such as density‐dependent losses. Although coexistence within functional groups was not stable, species co‐occurred for a very long time. Indeed, in the absence of stabilizing niche differences, equalizing mechanisms can reduce fitness differences and thus slow down but not prevent competitive exclusion in the long run (Chesson 2000). Furthermore, stabilizing mechanisms acting within functional groups need only to be small in order to allow coexistence and might be easily realized in natural systems through higher dimensional trade‐offs (Clark et al. 2010).

When trait adaptation was sufficiently high and restricted to species‐specific limits, we observed both equilibrium and nonequilibrium dynamics, which suggests that trait adaptation can stabilize coexistence via unique shifts and via ongoing redistribution of trait values. At equilibrium, coexistence is enabled without further trait adjustments (e.g., evolution) whereas ongoing trait changes promote coexistence due to a mutual interplay between biomass and trait dynamics (i.e., eco‐evolutionary dynamics or biomass–trait feedbacks). Hence, when predator and prey biomasses oscillated different trait values were favored at different times. As all species continuously adapted their trait values in response to selection the trait oscillations (ongoing coadaptation) were directly and inseparably related to the cycles in predator–prey biomasses. This is in line with findings from an experimental system of two competing plant species (Lankau and Strauss 2007) where coexistence was based on frequency‐dependent selection (Vasseur et al. 2011). In this case, trait adaptation and species diversity generated a feedback loop that maintained each other.

Depending on the current selection pressure trait divergence or trait convergence dominated the trait changes within the prey community leading to out‐of‐phase cycles between the trait values of more and less edible prey species in our model. For example, a dominance of selective predators selected for lower edibility within well edible and higher edibility within less edible species giving rise to trait convergence. In contrast, a dominance of rather nonselective predators promoted character divergence within the prey community as well edible prey species changed their edibility toward higher values and less edible prey species toward lower trait values. Therefore, two strategies temporally emerged within the prey community, either becoming defended or growing faster giving rise to temporal changes in niche and fitness differences (cf. Appendix S4).

Hence, the recurrent convergence and divergence of species' traits cause an interesting interplay between equalizing and stabilizing mechanisms as trait distances change: When traits are similar, species have similar fitness but low niche differences and vice versa. Thus, when stabilizing niche differences (here differences in grazing pressure) weakened, trait and thus fitness differences (here in intrinsic growth rates) decreased reducing the risk of competitive exclusion. Conversely, trait distances among species increased if stabilizing mechanisms were strong, compensating for reduced equalizing mechanisms. This way, the contribution of equalizing and stabilizing mechanisms was time‐dependent in systems with nonequilibrium dynamics. Our result is in line with recent findings of trait convergence toward a single strategy or trait divergence toward complementary strategies under competition for nutritionally essential (Macarthur and Levins 1967; Abrams 2000; Fox and Vasseur 2008) or substitutable (Lundberg and Stenseth 1985; Abrams 2000; Vasseur and Fox 2011) resources. However, these studies considered the long‐term behavior of the trait dynamics whereas our model reveals that trait convergence or divergence may vary temporally as a result of a biomass–trait feedback. Hence, based on the precondition that stable coexistence requires intraspecific competition to be on average larger than interspecific competition but not at every moment in time (cf. Vasseur et al. 2011), coexistence is promoted by the species potential to be sometimes more similar (neutral theory) or different (niche theory).

### Large and fast trait adaptation promoted neutrally stable coexistence – Region C2

For larger ranges of trait adaptation (*w > 0.3*), a transition from stable (niche differentiated) to rather neutrally stable (equalized) species coexistence occurred. Systems falling within this transition zone were usually marked by irregular biomass dynamics. The relatively low final richness and high sensitivity to environmental noise suggest a reduction in stabilizing mechanisms. However, for very high values of *w* the species shared a common range of trait adaptation allowing them to dynamically cluster into a single or a few different functional groups. In our model, at least two specialized prey or predator strategies emerged, suggesting the maintenance of stabilizing mechanisms based on self‐organized niche partitioning. Interestingly, the number of functional groups within the prey community usually differed from the one within the predator community giving rise to supersaturated coexistence, that is, the number of coexisting species exceeds the number of limiting resources. For example, in one case, only one prey strategy was supported in the long run whereas two strategies, that is, being either highly selective or nonselective, emerged within the predator community. The two predator functional groups coexisted by specializing on prey differing in their edibility and the oscillations in the trait values of the single prey functional type provided temporal opportunities for them to succeed.

Hence, the potential for trait adaptation gives rise to biomass–trait feedbacks that enable supersaturated coexistence. This directly corresponds to the generally debated importance of internally driven fluctuations in resource and consumer densities for maintaining species‐rich communities (Huisman and Weissing 1999; Huisman et al. 2001). Biomass fluctuations promoted coexistence in consumer–resource models where two consumers differed in their functional responses and competed for a common resource (Armstrong and McGehee 1980; Abrams and Holt 2002) and in a predator–prey model where the predator grazed on a genetically variable prey species (i.e., two specialists) and a phenotypic plastic prey species (i.e., the generalist) (Yamamichi et al. 2011). In these examples, a dominant competitor altered the environment in a way that allowed the other competitor to recover. For example, specialist consumers may promote cyclic predator–prey dynamics, enabling invasion by the generalist consumer which is superior when the prey composition fluctuates (Abrams 2006a,c; Holt et al. 2013).

Up to now, several studies emphasized that supersaturated coexistence is very sensitive to the chosen parameter values (Schippers et al. 2001), likely unstable (Roelke and Eldridge 2008) and that complex dynamics are not a likely mechanism to maintain high levels of genetic diversity (Jones et al. 2009). We challenge this point of view by showing that trait adaptation may enable stable supersaturated species coexistence in a wide parameter space by providing novel stabilizing and equalizing effects based on trait fluctuations. This finding is supported by our sensitivity analysis as neither changes in bottom‐up (*K*) nor top‐down control (*d*) altered the general patterns (cf. Appendix S3). Therefore, it is the inherent flexibility of species that makes their coexistence robust against environmental fluctuations. Indeed, low levels of environmental noise did not destabilize coexistence. Hence, studies such as Huisman and Weissing (1999) that show the potential for coexistence given the right parameter combinations are conservative, as trait adaptation is ubiquitous in natural systems and would find these and possibly more configurations that succeed. In summary, trait adaptation may enable species coexistence even if the number of limiting factors is smaller than the number of species and thus may contribute to resolve the paradox of the plankton (cf. Appendix S5).

## Conclusions

We used an innovative multispecies predator–prey model that allowed for simultaneous coadaptation among all prey and predator species. The model was parameterized for a distinct planktonic system but has very general properties. We demonstrate that the naturally ubiquitous but so far mostly neglected trait adaptation strongly increases the number of coexisting species, in particular when realistically restricted to species‐specific limits. Both niche differences and neutrality jointly acted to maintain species‐rich communities. Coadaptation among prey or predators yielded functional groups as species formed clumps along the trait axes. This reduced fitness differences among species of the same functional group while maintaining niche differences between species of different functional groups. Hence, species coexistence may arise from both, high trait similarities (resulting in ecological equivalence) and dissimilarities (resulting in niche differences) among species. In addition, coadaptation resulted in an ongoing convergence and divergence of species traits giving rise to a time‐dependent balance between equalizing and stabilizing mechanisms. In contrast to previous studies, the emergent feedback between biomass and trait fluctuations enabled supersaturated coexistence for a broad range of potential trait adaptation and parameters.

We conclude that the mismatch between the naturally observed species richness and theoretical predictions partly arises from assigning too rigid, temporally invariant mean values to the species' traits that underlie theory. Accepting the potential for trait changes as actual properties of natural systems allows to explain stable or supersaturated species coexistence for a broad range of environmental conditions. Hence, trait adaptation may be an important reason for the empirical evidence of high species richness in both aquatic and terrestrial systems.

## Conflict of Interest

None declared.

## Supporting information


**Appendix S1.** *v* scales the time‐scale of trait dynamics relative to population dynamics.
**Appendix S2.** Modelling trait adaptation within species‐specific limits.
**Appendix S3.** Sensitivity analysis.
**Appendix S4.** Coadaptation within and among trophic levels jointly promotes coexistence.
**Appendix S5.** Trait adaptation promotes stable supersaturated coexistence.
