# Supplementary material for: Trait adaptation promotes species coexistence in diverse predator and prey communities
Source: Ecol Evol. 2016 May 23;6(12):4141–59. doi: 10.1002/ece3.2172 (PMC4972238; doi:10.1002/ece3.2172)
Supplement: Supplementary file 1 — Appendix S1. v scales the time‐scale of trait dynamics relative to population dynamics. Appendix S2. Modelling trait adaptation within species‐specific limits. Appendix S3. Sensitivity analysis. Appendix S4. Coadaptation within and among trophic levels jointly promotes coexistence. Appendix S5. Trait adaptation promotes stable supersaturated coexistence. [file ECE3-6-4141-s001.docx]

**Appendix A: *v* scales the time-scale of trait dynamics relative to population dynamics**

To evaluate the influence of *v* on the relative time scale of the trait (evolutionary) and biomass (ecological) dynamics we extended the Geber-Price-Method (Hairston et al. 2005; Collins & Gardner 2009; Norberg et al. 2012) to multi-species predator-prey systems. This allows us to partition the relative contributions of species sorting (i.e. relative changes in biomasses due to direct or apparent competition), ecological interactions (i.e. absolute changes in biomasses due to growth or grazing losses) and adaptive evolution (or phenotypic plasticity) to the community’s aggregate properties such as the biomass-weighted community-wide mean per-capita net growth rate $\bar{R}_{P}$ of the prey. For each point in time, t, we can calculate $\bar{R}_{P}=\sum_{i=1}^{S} p_{i}\cdot R_{P_{i}}$where$p_{i}=P_{i}/\sum_{k=1}^{S} P_{k}$and$R_{P_{i}}=dP_{i}/P_{i}dt$ are the relative biomass and per capita net growth rate of the i-*th* prey species. Since$R_{P_{i}}=R_{P_{i}}\left[P_{1}...P_{S},C_{1'}...C_{S},\phi_{1}...\phi_{S},\omega_{1}...\omega_{S}\right]$depends on the biomasses and trait values of all prey and predator species $dR_{P_{i}}/dt$ can be decomposed into four parts:

$$\frac{dR_{P_{i}}}{dt}=\sum_{k=1}^{S} \frac{\partial R_{P_{i}}}{\partial P_{k}}\cdot\frac{\partial P_{k}}{\partial t}+\sum_{j=1}^{S} \frac{R_{P_{i}}}{\partial C_{j}}\cdot\frac{\partial C_{j}}{\partial t}+ \sum_{k=1}^{S} \frac{\partial R_{Pi}}{\partial\phi_{k}}\cdot\frac{\partial\phi_{k}}{\partial t}+\sum_{j=1}^{S} \frac{\partial R_{P_{i}}}{\partial\omega_{j}}\cdot\frac{\partial\omega_{j}}{\partial t}$$

As a result, the rate of change of the community-wide mean per capita net growth rate can also be decomposed into parts:

Using eq. *7* and eq. *8* (cf. methods), the relative time scale of the trait and biomass dynamics is given by:

$\underbrace{\left(v\cdot\sum_{i=1}^{S} \left[p_{i}\cdot\left(\sum_{k=1}^{S} \frac{\partial R_{P_{i}}}{\partial\phi_{k}}\cdot\frac{\partial R_{P_{i}}}{\partial\phi_{i}}+\sum_{j=1}^{S} \frac{\partial R_{P_{i}}}{\partial\omega_{j}}\cdot\frac{\partial R_{C_{j}}}{\partial\omega_{j}} \right)\right]\right)}_{\text{Adaptive} \text{evolution} \text{or} \text{phenotypic plasticity}}/\left(\underbrace{\sum_{i=1}^{S} \left[\frac{\partial p_{i}}{\partial t}\cdot R_{P_{i}}\right]}_{\text{Species sorting}} +\underbrace{\sum_{i=1}^{S} \left[p_{i}\cdot\left(\sum_{k=1}^{S} \frac{\partial R_{P_{i}}}{\partial P_{k}}\cdot\frac{\partial P_{k}}{\partial t}+\sum_{j=1}^{S} \frac{\partial R_{P_{i}}}{\partial C_{j}}\cdot\frac{\partial C_{j}}{\partial t} \right)\right]}_{\text{Ecological interactions}}\right)$Hence, *v* scales the relative time-scale of trait and biomass dynamics, and thus any increase in *v* will increase the rate of change of the species’ trait values relative to changes in their biomasses. For simplicity, we dropped the boundary function *B* from the expression above.

**Appendix B: Modelling trait adaptation within species-specific limits**

To describe the temporal development of the species’ mean trait values, $\bar{x}$, we adopted a general formula proposed by Abrams (2010) that is rooted in quantitative genetics:

The function *v* scales the rate of trait adaptation relative to the species’ population dynamics, *R* is the species’ per capita net growth rate and *B* is the boundary function representing non-adaptive trait changes that restrict the trait values to the ecologically feasible trait range. The sign denotes the evaluation of the species’ fitness-gradient ∂R/∂x at its mean trait value. Although this approach has been used primarily for traits with a genetic basis and thus for the representation of adaptive evolution (Lande 1982; Taper and Case 1985; Dieckmann and Law 1996), it may also be used to account for changes in the mean trait value via adaptive phenotypic plasticity (Abrams 2010; Mougi et al. 2011; Mougi and Iwasa 2011).

When considering trait changes within species-specific limits, the rate of change of $\bar{x}$has to slow down and finally approach zero when it approaches the extremes. This has frequently been achieved by making *v* itself dependent on $\bar{x}$(Abrams 1999; Tien and Ellner 2012). However, $\bar{x}$only uniquely determines the variance of the trait distribution when trait changes are based on shifts in the relative abundance of two extreme phenotypes. Nevertheless, since we consider a continuous quantitative trait, we assumed *v* to be constant and thus independent of $\bar{x}$ (cf. Schreiber et al. 2011; Vasseur et al. 2011). Hence, we restricted the trait range by including a boundary function *B* in eq. B1 that accounts for non-adaptive trait changes following Abrams and Matsuda (2004) (cf. eq. 10). It depends on both $\bar{x}$ and *v* satisfying the properties proposed by Abrams (2010): it is large and positive (or negative) when $\bar{x}$ approaches its minimum (or maximum) and it is very small for intermediate trait values. In accordance with theoretical studies of evolutionary biology (Saloniemi 1993) we slightly modified the framework of Abrams (2010) to make *B* dependent on *v*. This is reasonable since species with high values of *v*, reflecting a large standing trait variation, already comprise a high share of phenotypes which perceive the constraints of the boundary of the ecologically feasible trait range. This slows down adaptation. Below we provide a mathematical derivation for such a boundary function.

Consider a continuous quantitative trait that is restricted to a finite range because of physical or biological limits. For example, prey species cannot be less than inedible or more than entirely edible restricting the biologically feasible trait range between 0 (0% edible) and 1 (100% edible). To reflect this, one may assume that a species’ fitness becomes increasingly negative when its trait value passes the biologically feasible extremes. This property is captured by the following general differential equation describing the per-capita rate of change of the biomass of a subpopulation *n* with a particular trait value *x*.

$1/n\left(x\right)\cdot dn(x)/dt=f(x,N)-g(x,N)+\vartheta(x) (B2)$where *f* and *g* are the gross growth rate and death rate and $N=\int n(x)dx$ the total biomass of the population. The function *ϑ* restricts changes in the mean trait values to the biologically feasible range by assuming *ϑ* to be (approximately) zero within this range and to become increasingly negative for trait values approaching or passing the extreme trait values. One such function for the interval [0, 1] is given by:

$\vartheta\left(x\right)=-\log\left(\sin\left(\pi x\right)\right)/\pi(B3)$Note that the rate of change of *N* and $\bar{x}$ are given by

Following the approach of quantitative genetics (Lande 1982; Abrams et al. 1993) we now assume normally distributed trait values with a low and constant additive genetic variance. This way, we can approximate eq. B4 by:

According to eq. B5 we get:

where *dN’/N’dt* corresponds to the species’ average per-capita rate of change that is based solely on its ecologically relevant gross growth and death processes. The second term of eq. B6 corresponds to the boundary function *B* which yields a large positive and negative slope of the fitness landscape around the minimum and maximum trait values of the ecologically feasible trait range. This way, *B* can be viewed as part of the whole fitness landscape (eq. B6), determining the range over which biological interactions may change the landscape. Hence, *v* influences how fast trait changes approach the boundaries but not the boundaries of the fitness landscape themselves. Building on eq. B3 a boundary function that limits trait adaptation to the biologically feasible range, i.e. [0, 1], is given by:

$B\left(v,x\right)=-v\cdot\tan\left(\pi/2\cdot\left(2\cdot x-1\right)\right) (B7)$Since we partly consider very small ranges of trait adaptation in our model in which $\bar{x}$ is already close to the extreme trait values, we added an exponent *s* to eq. 10 (main text) to make our boundary function more stiff, thereby reducing confounding effects between $\bar{x}$ and *B* in these cases. Hence, with $B\left(v,x\right)=v\cdot\partial\vartheta(x)/\partial x$ we included a limiting function $\vartheta(x)$ into eqns. 7 and 8 describing trait adaptations of the prey and predator species. We dropped this term from eqns. 1 and 2 since it has a negligible effect on the rate of biomass change over the vast majority of the trait range but would slow down numerical integration.

**Appendix C: Sensitivity analysis**

To test the robustness of our results with respect to parameter changes we conducted numerical simulations comprising a full-factorial combination of 11 carrying capacities (*K*ϵ[5,6,…,15]) and 11 death rates of the predators (*d*ϵ[0.1,0.11,…,0.2]) simulating changes in the bottom-up regulation and top-down control of the prey. We analyzed 3 different predator-prey systems differing in the species’ range of trait adaptation (*w*ϵ[10^-2^, 10^-1^, 10^0^]).

The influence of *w* on species coexistence was largely independent of the parameters varied (Fig. C1). For *w*=10^-2^ usually one prey and one predator species survived at the end of the simulation independent of *K* and *d*, except for a combination of low values of *K* and low or very high values of *d*. At very high values of *d*, predators suffered from low food availability and high mortality leading to their exclusion and neutrally stable coexistence of some prey species at their carrying capacity. Coexistence was neutrally stable since any small perturbation would promote the replacement of the less edible species by the most edible species which are the competitive superior in the absence of predators. In contrast, low values of *d* often prevented selective predators from exhibiting strong biomass declines during periods of starvation reducing their risk of extinction.

For *w*=10^-1^ and *w*=1 final richness was always high except for a combination of high values of *K* and low values of *d*. In this case, an increased top-down control and a synchronization of the prey species by non-selective predators destabilized population and community dynamics. As a result, many prey and predator species were excluded. Hence, coadaptation strongly increased the parameter range where generalist and specialist consumers can coexist which is in line with findings from Abrams (2006) considering trait adaptation only at one trophic level.

Despite the positive influence of trait adaptation on species coexistence for a wide range of parameters our results also indicate that we can expect a decrease in final richness of prey and predator species when increasing or decreasing *K.* This leads to a humped-shaped dependency of final richness on the carrying capacity *K* (Fig. C1, B; cf. Leibold 1996). For very low values of *K* prey biomass is frequently too low to support the food demands of the less selective predators leading to their extinction. As a consequence, the system will get locked in a state of inedible prey species promoting further extinctions of more edible prey species and the more selective predators. In contrast, very high values of *K* strongly improve the performance of less selective predators compared to more selective ones initially promoting the exclusion of more selective predators and ultimately of less edible prey species because the costs for their defense do not pay off.

The general framework underlying our model was also analyzed with respect to the role of various other parameters including the maximum growth rate (*r_max_*) of the prey, the maximum grazing rate (*g*) of the predators and the parameters *b* and *c* determining the feeding interactions between predator and prey species (Tirok and Gaedke 2010; Tirok et al. 2011; Bauer et al. 2014). The results from their sensitivity analyses are assumed to hold for our case as well.


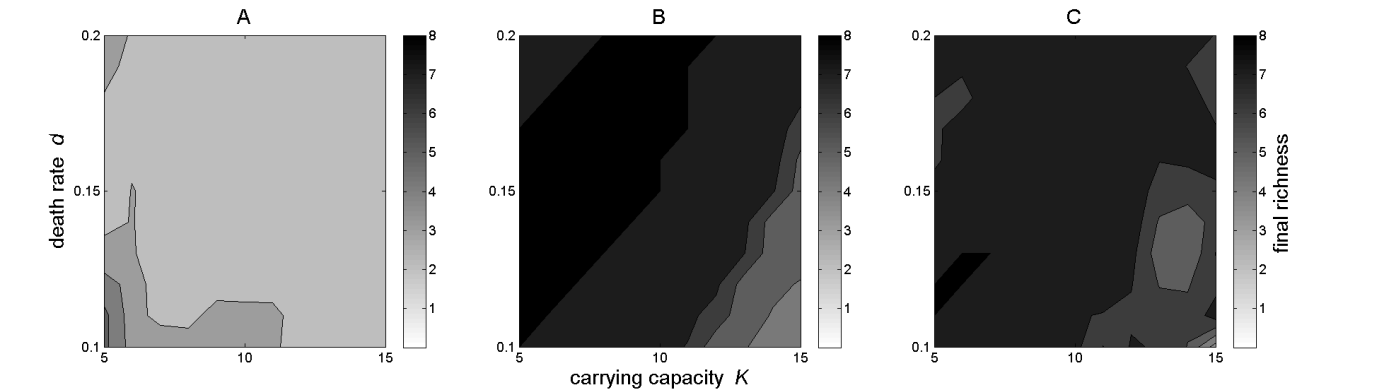


**Figure C1.** Final richness summed over the prey and predator communities depending on the prey’s carrying capacity (*K*) and the predator’s death rate (*d*) for S=4, *v*=10^-1.5^ and *w*=10^-2^ (A), *w*=10^-1^ (B) and *w*=10^0^ (C).

**Appendix D: Coadaptation within and among trophic levels jointly promotes coexistence**

We provide here a more detailed evaluation of the mechanisms underlying species coexistence in our multispecies predator and prey communities in region C1 of our simulation results, combining numerical simulations and analytical considerations.

First, we evaluated the importance of coadaptation within and among trophic levels for coexistence numerically by analyzing our model for cases where only a subset of prey or predator species were able to adapt their trait values in response to selection. We simulated all possible combinations with 0 to 8 adaptive species for 25.000 time steps which was usually enough to reach quasi-equilibrium. We further compared the effects of adaptive to non-adaptive trait changes by considering situations where the trait values changed only randomly irrespective of what the other species were doing. We modeled non-adaptive trait changes by modifying equations 7 and 8 in the following way:

$\begin{matrix} \frac{d\phi_{i}}{dt}=v\cdot\left(n(0,1)+B\left(\phi_{i},\Phi_{i}\right)\right) (D1) \\ \frac{d\omega_{j}}{dt}=v\cdot\left(n(0,1)+B\left(\omega_{j},\Omega_{j}\right)\right) (D2) \end{matrix}$where the term n(0,1) represents random numbers that were drawn independently from a normal distribution with mean and standard deviation equal to 0 and 1 for each time step and differential equation prior to numerical integration. Hence, trait changes followed additive white noise.

Increasing the number of adaptive species increased final richness in our model (Fig. D1; Fig. D2). In particular, final richness was maximized when at least 6 species and thus at least 2 prey and predator species were adaptive irrespective of the species identities. This suggests that coadaptation within and among trophic levels interacted to promote species-rich communities in our model. In contrast, non-adaptive trait changes did not increase final richness but often promoted the exclusion of all species (Fig. D1; Fig. D2).

We further disentangled the relative importance of coadaptation within and among trophic levels on coexistence. First, to evaluate the role of coadaptation within a trophic level, we allowed only the prey or the predators to change their traits. Second, to evaluate the effect of coadaptation between the two trophic levels, we allowed trait adaptation in both the prey and the predator communities, but all prey and all predator species had to change their traits in concert and thus in response to a common selection pressure. This was achieved by changing equations 7 and 8 in the following way:

$\begin{matrix} \frac{d\phi_{i}}{dt}=v\cdot\left(\sum_{k=1}^{S} \left(p_{k}\cdot\frac{\partial R_{P_{k}}}{\partial\phi_{k}}\right)+B\left(\phi_{i},\Phi_{i}\right)\right) (D3) \\ \frac{d\omega_{j}}{dt}=v\cdot\left(\sum_{k=1}^{S} \left(p_{k}\cdot\frac{\partial R_{C_{k}}}{\partial\omega_{k}}\right)+B\left(\omega_{j},\Omega_{j}\right)\right) (D4) \end{matrix}$Hence, all species change their trait values according to the biomass-weighted (*p_k_*) average of the species-specific fitness gradients thereby preventing coadaptation within a trophic level. To ensure synchronous trait dynamics during the entire simulation time we started all simulations with trait values equal to *φ_i_=Φ_i_-w∙(Φ_i_-0.5)* for prey and *ω_j_=Ω_j_-w∙(Ω_j_-0.5)* for predators, respectively.

Our results show that coadaptation within and among trophic levels similarly promoted final richness. When only the prey or the predators could adapt final richness decreased from 8±0 (mean±std; n=25) to 5.5±0.9 or 5.8±0.7, respectively (Fig. D3 A, B). When coadaptation within a trophic level was absent final richness decreased to 4.8±1.5 (Fig. D3 C).

The numerical results can be more clearly understood by considering the equations underlying the trait and biomass dynamics. The partial derivatives given in eq. 7 and eq. 8 of the main text denote the fitness gradients of the species evaluated at their respective mean trait values. Their analytical expressions provided below show how the feedback between biomass and trait dynamics promotes species coexistence.

The fitness gradient of prey species *i* is given by (for parameters see Table 1):

$\frac{\partial R_{P_{i}}}{\partial\phi_{i}}=\left(1-\frac{\sum_{z=1}^{S} P_{z}}{K}\right)\cdot\left(r_{\max}-r_{\min}\right)-\sum_{j=1}^{S} \frac{g_{\max}\cdot C_{j}\cdot b\cdot q\left(\phi_{i},\omega_{j}\right)\cdot\left(\sum_{z=1}^{S} q\left(\phi_{z},\omega_{j}\right)\cdot P_{z}-\left(q\left(\phi_{i},\omega_{j}\right)\cdot P_{i}\right)+H\left(\omega_{j}\right)\right)}{\left(e^{b\cdot\left(\phi_{i}-c\cdot\omega_{j}\right)}+1\right)\cdot\left(\sum_{z=1}^{S} q\left(\phi_{z},\omega_{j}\right)\cdot P_{z}+H\left(\omega_{j}\right)\right)^{2}} (D5)$In the presence of strong grazing pressure by non-selective predators the second term of eq. D5 will be negligible. Hence, changes in the mean trait values of the prey will be mainly driven by the first term promoting shifts towards higher edibility increasing the prey’s competiveness. In contrast, a high prey biomass close to the carrying capacity *K* combined with a substantial predation pressure of selective predators will favor trait changes within prey species towards lower edibility based on the second term of eq. D5. This reduces the grazing pressure on the prey buffering them from extinction.

The fitness gradient of predator species *j* is given by:

$\frac{\partial R_{C_{j}}}{\partial\omega_{j}}=e\cdot g_{\max}\cdot\left[\frac{P_{i}\cdot\frac{-c\cdot b\cdot q\left(\phi_{i},\omega_{j}\right)}{\left(e^{b\cdot\left(\phi_{i}-c\cdot\omega_{j}\right)}+1\right)}}{\left(\sum_{z=1}^{S} q\left(\phi_{i},\omega_{j}\right)\cdot P_{z}+H\left(\omega_{j}\right)\right)}-\frac{P_{i}\cdot q\left(\phi_{i},\omega_{j}\right)\cdot\left(\sum_{z=1}^{S} \frac{-c\cdot b\cdot q\left(\phi_{i},\omega_{j}\right)}{\left(e^{b\cdot\left(\phi_{i}-c\cdot\omega_{j}\right)}+1\right)}\cdot P_{z}-\left(H_{\max}-H_{\min}\right)\right)}{\left(\sum_{z=1}^{S} q\left(\phi_{i},\omega_{j}\right)\cdot P_{z}+H\left(\omega_{j}\right)\right)^{2}}\right] (D6)$In the presence of well edible prey the second term of eq. D6 will promote changes in the traits of the predators towards higher selectivity. This arises from a dominant influence of the slope of the trade-off between half-saturation constant and selectivity. In contrast, if less edible prey species dominate the prey community the derivative of the preference function in both terms of eq. D6 will jointly favor trait changes towards lower values of ω. This increases the grazing pressure on the dominant prey thereby reducing periods of food shortage. Hence, coadaptation among prey and predators gives rise to negative frequency and density dependence promoting stable species coexistence.

In addition, considering the equations underlying the biomass dynamics in the prey community reveals that coadaptation among prey allows a temporal convergence and divergence of traits that gives rise to temporal changes in niche and fitness differences among prey species. The rate of change of the i-*th* prey species biomass can be expressed as:

$$\frac{dP_{i}}{dt}=r\left(\phi_{i}\right)\cdot\left(1-\sum_{z=1}^{S} P_{z}/K\right)\cdot P_{i}-\eta\left(\phi_{i}\right)\cdot P_{i} =\left(r\left(\phi_{i}\right)-\eta\left(\phi_{i}\right)\right)\cdot\left(1-\sum_{z=1}^{S} P_{z}/K\cdot\left(1-\frac{\eta\left(\phi_{i}\right)}{r\left(\phi_{i}\right)}\right)\right)\cdot P_{i} \left(D7\right)$$

with$\eta\left(\phi_{i}\right)=g_{\max}\cdot\sum_{j=1}^{S} q_{i,j}/\left(\sum_{z=1}^{S} q_{z,j}\cdot P_{z}+H_{j}\right) (D8)$

being the per capita death rate of the i-*th* prey species caused by grazing (cf. eq. 1). This formulation shows how trait adaptation can increase a prey species’ effective capacity and thus its (direct or apparent) competitive ability either by increasing its growth rate or reducing its death rate giving rise to environment-dependent temporal convergence or divergence of prey species traits (Fig. D3 A). A dominance of highly selective predators promotes shifts towards lower and higher trait values in more and less edible prey species, respectively (trait convergence). The opposite and thus divergence among prey species’ traits happens when moderately selective species dominate the predator community.


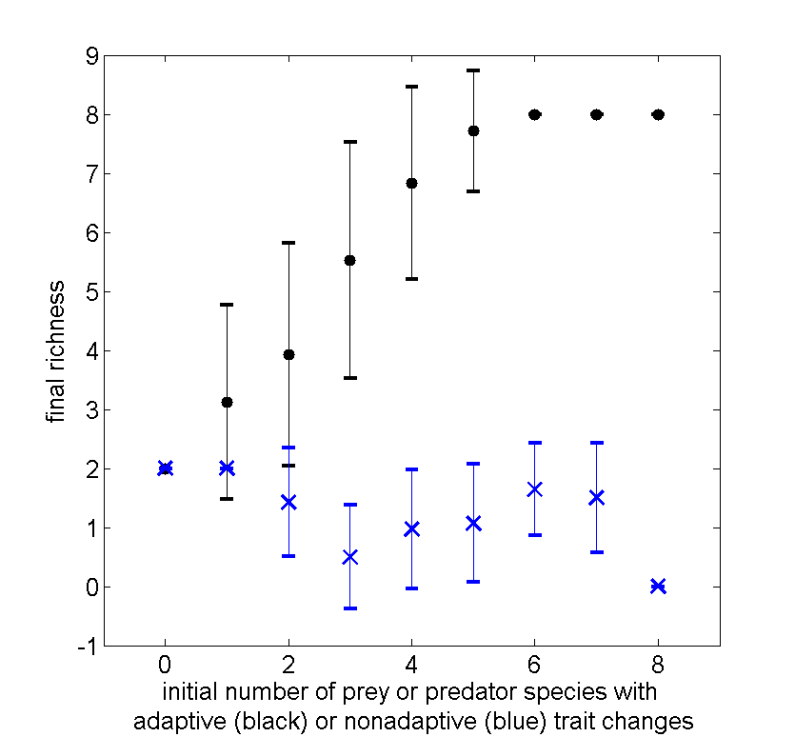


**Figure D1** Average and standard deviation of summed final prey and predator richness in relationship to the initial number of species which are able to adjust their mean trait values in response to selection (black dots) or which exhibit random trait changes (blue crosses).


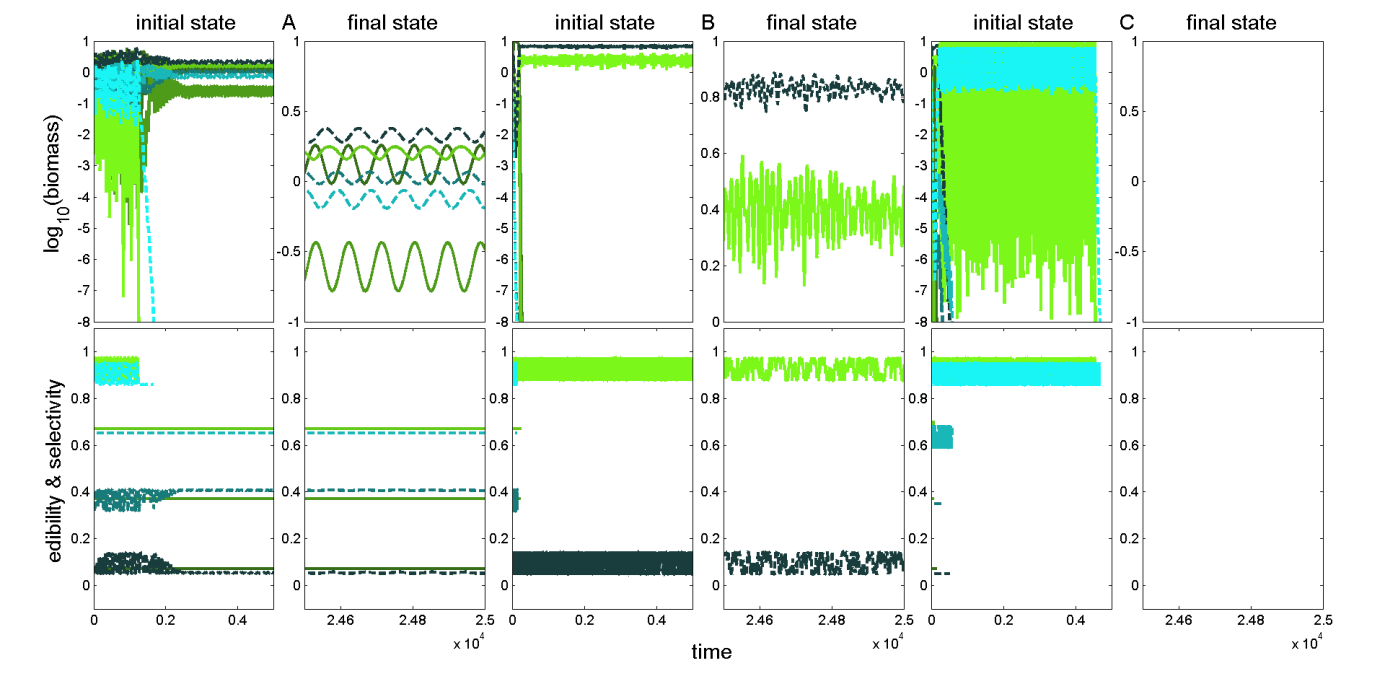


**Figure D2** Biomass (top) and trait dynamics (bottom) for intermediate values of the range (*w*=0.1) and speed (*v*=10^-1.5^) of trait adaptation for adaptive (A) or non-adaptive (B, C) trait changes within 4 species, showing the first 5000 time steps (left panels) and last 500 time steps (right panels) for four prey (green) and four predator species (blue). Initial edibility and selectivity increase from dark to light shades of colors (cf. Fig. 2). In C, all prey or predator species were excluded.


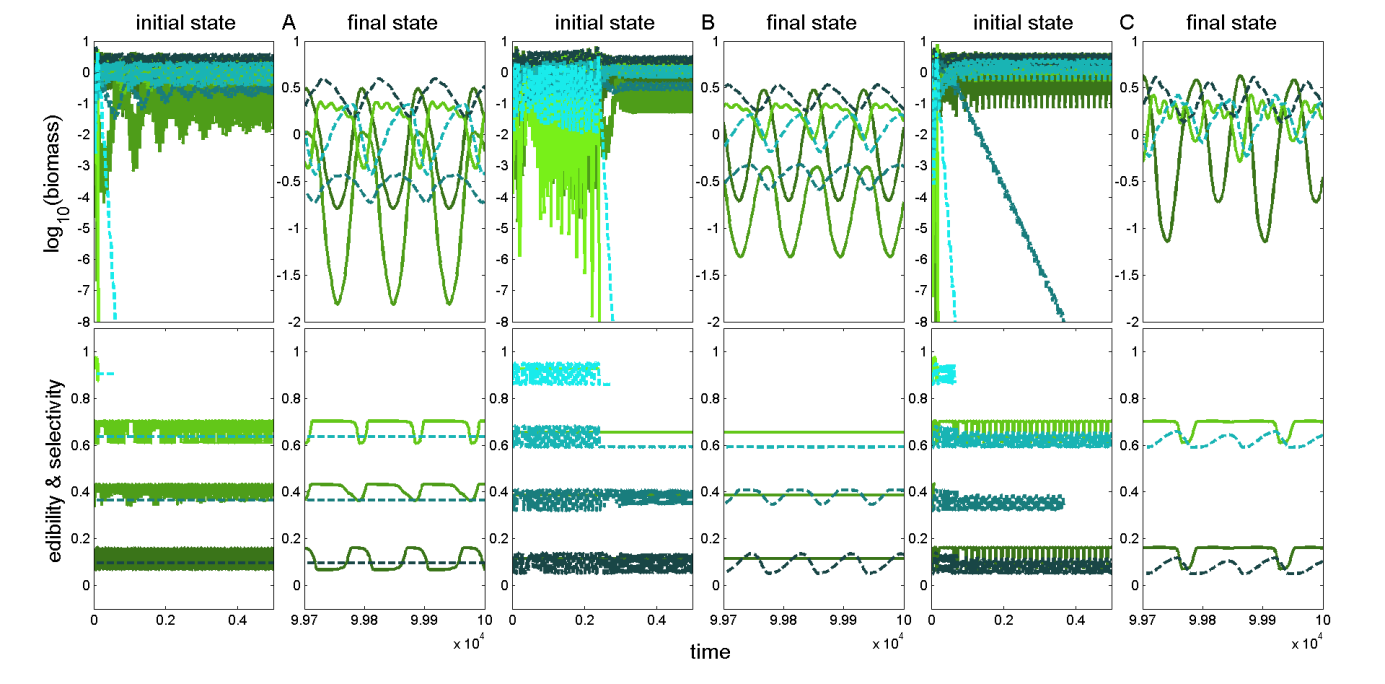


**Figure D3** Biomass (top) and mean trait dynamics (bottom) for intermediate values of the range (*w*=0.1) and speed (*v*=10^-1.5^) of trait adaptation allowing trait changes only within A) the prey or B) the predators and C) only between the prey and predators (trait changes within predator and prey communities synchronized), showing the first 5000 time steps (left panels) and last 300 time steps (right panels) for four prey (green) and four predator species (blue). Initial edibility and selectivity increase from dark to light shades of colors (cf. Fig. 1).

**Appendix E: Trait adaptation promotes stable supersaturated coexistence**

The paradox of the plankton questions how a large number of phytoplankton species can coexist in a rather homogeneous environment while all compete for the same few limiting resources (Hutchinson 1961). We show that trait adaptation may help to resolve this paradox as it strongly promotes supersaturated coexistence, where the number of species exceeds the number of limiting factors.

In the main text, we presented simulation results using an equal number of initial prey and predator species. Without trait adaptation (region E1, cf. Fig. 3), we never observed supersaturated coexistence (Fig. E1, A). In contrast, when species were sufficiently able to adapt their trait values in response to selection the final richness in one trophic level often exceeded the richness in the other one. In regions E2 and C2 (cf. Fig. 3) 25% and 33% of the simulations showed such a supersaturated coexistence (Fig. E1, B, D).

Furthermore, starting with the same initial richness of prey and predator species may strongly underestimate the potential for trait adaptation to generate supersaturated coexistence. For example, in region C1 all prey and predator species were usually maintained preventing the possibility of supersaturated coexistence (Fig. E1, C). Hence, we present here additional simulations with an unequal initial number of prey (S_prey_=6) and predators (S_predators_=2) for *v*=0.1, 25 different initial conditions and 21 different values of *w* ([10^-2^,10^-1.9^,…,10^0^]). Supersaturated prey coexistence occurred from w=0.1 onwards and final prey richness strongly increased with *w* (Fig. E1, E; Fig. E2). In the absence of trait adaptation, two prey species with similar trait values coexisted as each of them was limited by a different predator (Fig. E2, A). In contrast, sufficiently large ranges of trait adaptation allowed for stable supersaturated coexistence of several functionally different prey species where each prey species was temporally limited by one of the two predators (Fig. E2, B). We obtained similar results when conducting the corresponding simulations with initially two prey and six predator species. In the absence of trait adaptation, usually one predator and one prey survived (Fig. E3, A). Supersaturated predator coexistence occurred from w=0.25 onwards where each predator species was temporally limited by one of the two prey species (Fig. E3, B).

In accordance with the results described in the main text, very large ranges of trait adaptation enabled prey and predators to cluster into functional groups thereby allowing a large number of prey species or predator species to coexist on a few limiting factors (Fig. E1; Fig. E2, C; Fig. E3, C). Hence, trait adaptation may strongly facilitate supersaturated coexistence in natural systems such as zoo- and phytoplankton communities.


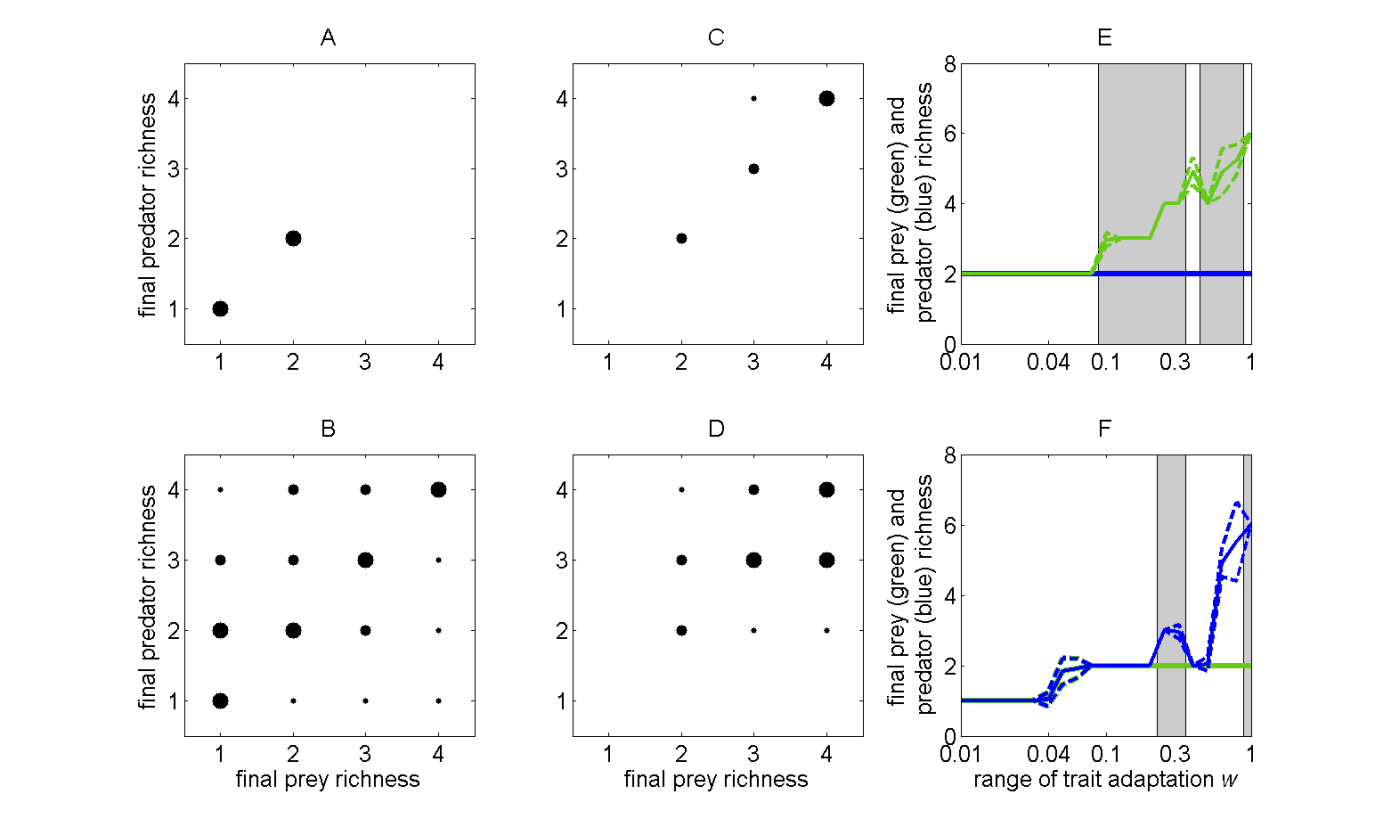


**Figure E1** A-D) Final prey versus final predator richness for parameter regions E1 (A), E2 (B), C1 (C) and C2 (D) (cf. Fig. 3 main text). The relative frequency is indicated by large (100%≥p>10%), intermediate (10%≥p>1%) or small dot sizes (p≤1%). E-F) Average (solid line) and standard deviation (dashed line) of final prey (green) and predator richness (blue) depending on the range of trait adaptation *w* for *v*=0.1 and initially 2 predator and 6 prey species (E), respectively 6 predator and 2 prey species (F). Grey shaded areas mark parameter regions of stable supersaturated prey (E) respectively predator (F) coexistence, that is prey and predator species do not exhibit long-term trends independent of the initial conditions.


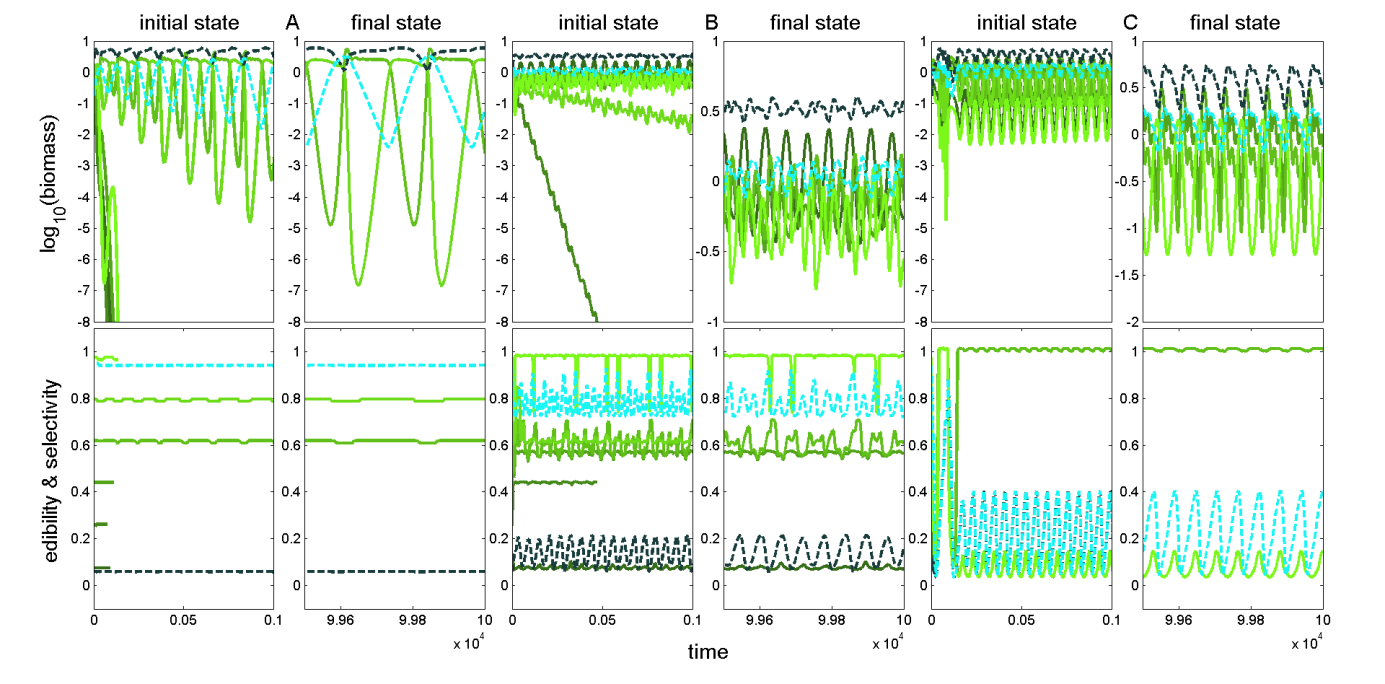


**Figure E2** Biomass (top) and mean trait dynamics (bottom) for moderate speed (*v*=10^-1^) and A) small (*w*=0.01), B) intermediate (*w*=0.25) and C) large (*w*=1) ranges of trait adaptation, showing the initial (left panels) and final (right panels) state for initially six prey (colored green) and two predator species (colored blue). Initial edibility and selectivity increase from dark to light shades of colors (cf. Fig. 1). Note the different scales for the two time periods because of differences in the amplitudes and periods of the oscillations during the transients and the final states.


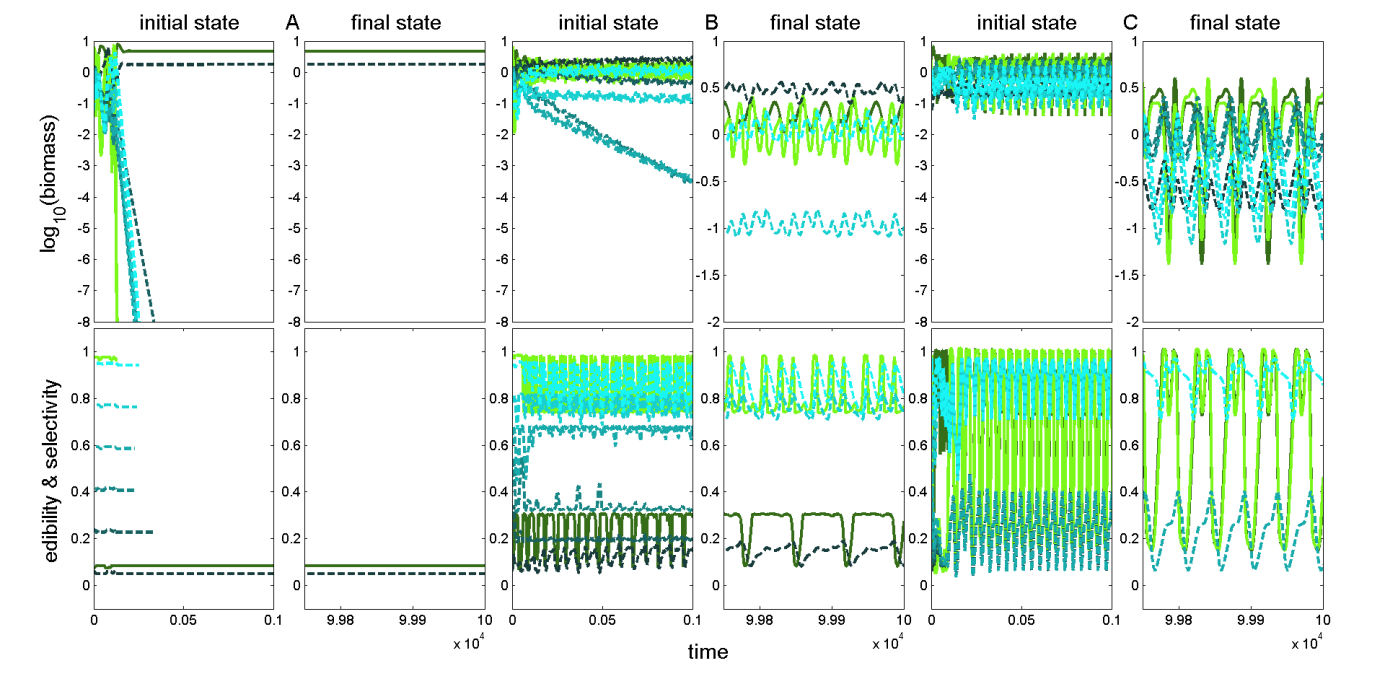


**Figure E3** Biomass (top) and mean trait dynamics (bottom) for moderate speed (*v*=10^-1^) and A) small (*w*=0.01), B) intermediate (*w*=0.25) and C) large (*w*=1) ranges of trait adaptation, showing the initial (left panels) and final (right panels) state for initially two prey (colored green) and six predator species (colored blue). Initial edibility and selectivity increase from dark to light shades of colors (cf. Fig. 1).

Literature Cited

Abrams, P. A. 1999. The adaptive dynamics of consumer choice. American Naturalist 153:83-97.

Abrams, P. A. 2006. The prerequisites for and likelihood of generalist-specialist coexistence. American Naturalist 167:329-342.

Abrams, P. A., Y. Harada, and H. Matsuda. 1993. On the Relationship between Quantitative Genetic and ESS Models. Evolution 47:982-985.

Abrams, P. A. and H. Matsuda. 2004. Consequences of behavioral dynamics for the population dynamics of predator-prey systems with switching. Population Ecology 46:13-25.

Abrams, P. A. 2010. Quantitative descriptions of resource choice in ecological models. Population Ecology 52:47-58.

Bauer, B., M. Vos, T. Klauschies, and U. Gaedke. 2014. Diversity, Functional Similarity, and Top-Down Control Drive Synchronization and the Reliability of Ecosystem Function. American Naturalist 183:394-409.

Collins S. and A. Gardner. 2009. Integrating physiological, ecological and evolutionary change: a Price equation approach. Ecology Letters 12:744-757.

Dieckmann, U. and R. Law. 1996. The dynamical theory of coevolution: A derivation from stochastic ecological processes. Journal of Mathematical Biology 34:579-612.

Hairston, N. G., S. P. Ellner, M. A. Geber, T. Yoshida, and J. A. Fox. 2005. Rapid evolution and the convergence of ecological and evolutionary time. Ecology Letters 8:1114-1127.

Hutchinson, G. E. 1961. The Paradox of the Plankton. American Naturalist 95:137-145.

Lande, R. 1982. A Quantitative Genetic Theory of Life-History Evolution. Ecology 63:607-615.

Leibold, M. A. 1996. A graphical model of keystone predators in food webs: Trophic regulation of abundance, incidence, and diversity patterns in communities. American Naturalist 147:784-812.

Mougi, A. and Y. Iwasa. 2011. Green world maintained by adaptation. Theoretical Ecology 4:201-210.

Mougi, A., O. Kishida, and Y. Iwasa. 2011. Coevolution of Phenotypic Plasticity in Predator and Prey: Why Are Inducible Offenses Rarer Than Inducible Defenses? Evolution 65:1079-1087.

Norberg, J., M. C. Urban, M. Vellend, C. A. Klausmeier, and N. Loeuille. 2012. Eco-evolutionary responses of biodiversity to climate change. Nature Climate Change: 1-5.

Saloniemi, I. 1993. A Coevolutionary Predator-Prey Model with Quantitative Characters. American Naturalist 141:880-896.

Schreiber, S. J., R. Burger, and D. I. Bolnick. 2011. The community effects of phenotypic and genetic variation within a predator population. Ecology 92:1582-1593.

Taper, M. L. and T. J. Case. 1985. Quantitative Genetic Models for the Coevolution of Character Displacement. Ecology 66:355-371.

Tien, R. J. and S. P. Ellner. 2012. Variable cost of prey defense and coevolution in predator-prey systems. Ecological Monographs 82:491-504.

Tirok, K., B. Bauer, K. Wirtz, and U. Gaedke. 2011. Predator-Prey Dynamics Driven by Feedback between Functionally Diverse Trophic Levels. Plos One 6.

Tirok, K. and U. Gaedke. 2010. Internally driven alternation of functional traits in a multispecies predator-prey system. Ecology 91:1748-1762.

Vasseur, D. A., P. Amarasekare, V. H. W. Rudolf, and J. M. Levine. 2011. Eco-Evolutionary Dynamics Enable Coexistence via Neighbor-Dependent Selection. American Naturalist 178:E96-E109.
